# Systematic Review and Meta-Analysis of Randomized Clinical Trials in the Treatment of Human Brucellosis

**DOI:** 10.1371/journal.pone.0032090

**Published:** 2012-02-29

**Authors:** Julián Solís García del Pozo, Javier Solera

**Affiliations:** 1 Department of Internal Medicine, Villarrobledo Hospital, Villarrobledo, Spain; 2 Department of Internal Medicine, Albacete University Hospital, Albacete, Spain; Sapienza University of Rome, Italy

## Abstract

**Background:**

Brucellosis is a persistent health problem in many developing countries throughout the world, and the search for simple and effective treatment continues to be of great importance.

**Methods and Findings:**

A search was conducted in MEDLINE and in the Cochrane Central Register of Controlled Trials (CENTRAL). Clinical trials published from 1985 to present that assess different antimicrobial regimens in cases of documented acute uncomplicated human brucellosis were included. The primary outcomes were relapse, therapeutic failure, combined variable of relapse and therapeutic failure, and adverse effect rates. A meta-analysis with a fixed effect model was performed and odds ratio with 95% confidence intervals were calculated. A random effect model was used when significant heterogeneity between studies was verified.

Comparison of combined doxycycline and rifampicin with a combination of doxycycline and streptomycin favors the latter regimen (OR = 3.17; CI95% = 2.05–4.91). There were no significant differences between combined doxycycline-streptomycin and combined doxycycline-gentamicin (OR = 1.89; CI95% = 0.81–4.39). Treatment with rifampicin and quinolones was similar to combined doxycycline-rifampicin (OR = 1.23; CI95% = 0.63–2.40). Only one study assessed triple therapy with aminoglycoside-doxycycline-rifampicin and only included patients with uncomplicated brucellosis. Thus this approach cannot be considered the therapy of choice until further studies have been performed. Combined doxycycline/co-trimoxazole or doxycycline monotherapy could represent a cost-effective alternative in certain patient groups, and further studies are needed in the future.

**Conclusions:**

Although the preferred treatment in uncomplicated human brucellosis is doxycycline-aminoglycoside combination, other treatments based on oral regimens or monotherapy should not be rejected until they are better studied. Triple therapy should not be considered the current treatment of choice.

## Introduction

Brucellosis is one of the most common zoonotic diseases world-wide [1] and it continues to be a health problem in developing countries. Despite the existence of effective treatments, it may be responsible for high morbidity [2], [3]. The most common treatments are combined doxycycline and rifampicin for 6 weeks [4], and doxycycline for 6 weeks combined with an aminoglycoside (primarily streptomycin or gentamicin) [5] over the first few days of treatment. However, there are still a number of obstacles to overcome, such as the need for parenteral administration of aminoglycosides, the danger of inducing rifampicin resistance in countries where tuberculosis poses a problem, treatment compliance in a disease in which symptoms disappear a few days after initiating treatment, the difficulty of patient follow-up in underdeveloped rural areas, and the relapses, which affect approximately 10% of the patients [5], [6]. Furthermore, ever since the first effective treatments against brucellosis appeared (based on combined sulphonamides or tetracyclines and aminoglycosides), antimicrobial combinations have been preferred over monotherapy, and alternative that has seldom been used and studied. On the other hand, brucellosis may be complicated by endocarditis, neurobrucellosis or osteoarticular infections such as spondylitis. These forms may require longer or more aggressive treatment than uncomplicated brucellosis, and they should be studied separately.

In 1990, Hall [7] published an extensive review of treatments for human brucellosis in which he pointed out that until then there had been only four comparative prospective randomized studies concerning the therapeutic options for brucellosis. Two of these studies were published by Ariza *et al.* in 1985 [8], [9], another by Acocella *et al.* in 1989 [10] and a further study by Colmenero *et al.* in 1989 [11]. In the past 25 years, the results of other clinical trials have been published, as well as various reviews and meta-analyses aimed at identifying the best treatment for brucellosis [12]–[14]. In the most recent of these papers, published in 2008 [14], Skalsky *et al.* recommended a combination of three drugs as the therapy of choice for human brucellosis (doxycycline, rifampicin for 6 to 8 weeks, and aminoglycoside for 7 to 14 days). This recommendation is based on two studies. One study, involved the comparison of 5 different therapeutic regimens in 102 patients with lumbar spondylitis [15] and concluded recommending streptomycin for 15 days and doxycycline and rifampicin for 45 days in these cases. However, in patients with focal complications of brucellosis such as brucella spondylitis, longer or more aggressive curative therapy may be necessary than in patients with uncomplicated brucellosis. The second study [16] compared amikacin for 7 days combined with doxycycline and rifampicin for 8 to 12 weeks vs. doxycycline and rifampicin also for a period of 8 to 12 weeks. The conclusions of this study were based on a lower treatment failure rate (as defined by the disappearance of fever and symptoms) associated with the triple therapy, with limited significance (p = 0.04, CI = 95%: 0.008–0.15) when compared with the doxycycline-rifampicin regimen. Specifically, 92.2% of patients were afebrile after two weeks of treatment with triple therapy, and 68% were afebrile with combined doxycycline-rifampicin, in sharp contrast to other studies [8], [17]–[22]. The study revealed no differences in terms of relapse (p = 0.4). Triple therapy makes treatment more complicated, increases costs and makes administration more difficult, especially in developing countries. These results illustrate that the need for better and inexpensive treatments remains. Further, combining data from such different forms of brucellosis as spondylitis and non-focal brucellosis may be inapropiate and could lead to erroneous conclusions.

The ideal treatment should be given orally, thus increasing compliance, and should not involve increased rates of relapse or treatment failure. In this paper, a critical review of published data will be presented in order to identify therapeutic regimens allowing effective and easy-to-use treatment, and a number of questions will be posed that must be examined in future studies. The aim is to determine which of the standard therapeutic approaches is the most advisable, whether there are any alternative approaches, whether triple therapy could be recommended as the best option in view of current data, whether monotherapy represents a valid choice, and what treatment duration should be recommended for non-focal brucellosis.

## Methods

### Search Strategy

A search was conducted for all studies assessing different antimicrobial regimens in the treatment of human brucellosis from 1985 until the present day. The studies included are those comprising cases of brucellosis identified by isolation of bacteria of the *Brucella* genus or by clinical and serological signs consistent with acute *Brucella spp* infection.

**Figure 1:**
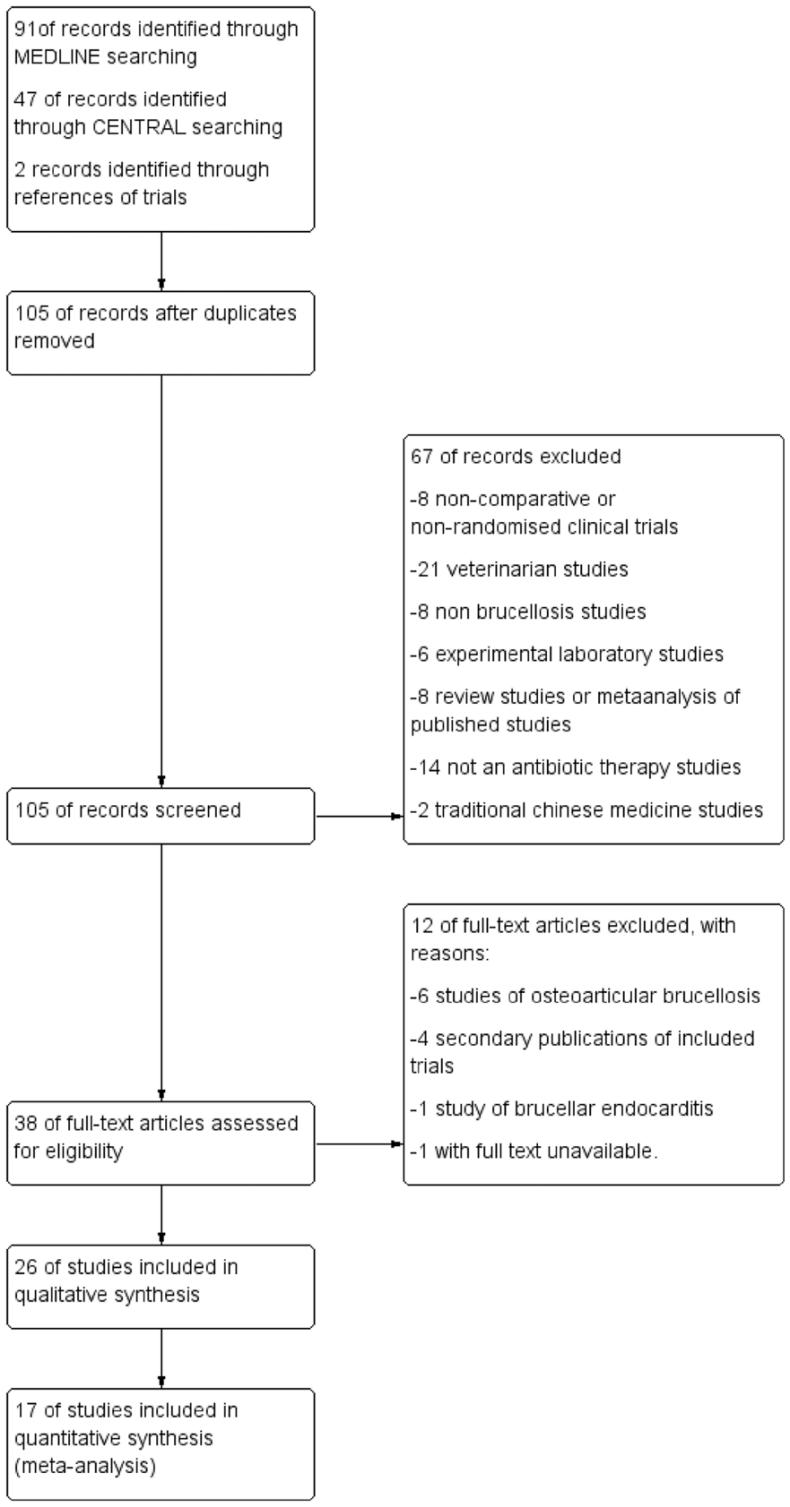
Study flow diagram.

The studies were identified by means of a MEDLINE search using the terms “*brucella*” or “*human brucellosis*”, and “*treatment*” or “*therapy*” and “*clinical trial*”. This search yielded 102 studies (January 2011), 52 of which were rejected because they were veterinary studies (21), lied outside the timeframe of the review (11), did not refer to brucellosis (8), were review articles (7) or were experimental laboratory studies(5). Of the 50 remaining studies, 12 were not studies of antimicrobial therapy, 8 were non-randomized, 6 involved investigation of osteoarticular brucellosis or brucellar endocarditis and 1 was a traditional Chinese medicine survey. The 23 remaining studies were randomized clinical trials in patients presenting acute brucellosis. One of these was a study published by Rodríguez Zapata *et al.* [23], which was excluded because the results are included in another and more extensive study [10]. Twenty two studies were thus selected. A search of the Cochrane Central Register of Controlled Trials (CENTRAL) was conducted for studies related to the term “brucellosis”, which resulted in the identification of two additional studies meeting the criteria for inclusion in our analysis. The bibliographic references of the selected articles were also examined in search of other possible publications not found in the above-mentioned databases (Text S1 and table S1). Study flow diagram is shown in figure 1.

### Selection criteria

Studies comparing two or more antibiotic treatment regimens in human brucellosis were analyzed. Only studies with fully characterized patients in terms of diagnosis, treatment administered, and duration and dosage of treatment were selected, and they also had to include a minimum follow-up period of six months. The analysis included studies of patients with acute brucellosis, and excluded studies performed solely on patients with focal osteoarticular brucellosis, neurobrucellosis or brucellar endocarditis. Congress abstracts were not included.

Studies without adequate details of the drugs, the doses or the duration of treatment, were excluded. Also excluded were the studies in which diagnostic criteria were not specified, which failed to meet the above-set criteria (with a follow-up of less than six months) (excluded studies are listed in table S2).

In addition, data in non-comparative studies were separately and complementarily searched, in order to obtain information of regimens not included in randomized comparative studies. Although these studies are cited in the initial description of each regimen, they were not included in the aforementioned statistical analysis, and the non-comparative nature of the test or study is indicated in each case.

An additional section with information collected from case series with 100 or more patients published over the past 10 years is also included, in which data concerning treatment and clinical outcome as well as information concerning the use of the regimens in the clinical practice is provided. Smaller patient series or individual case studies were not considered.

### Outcome measures

The main parameters considered in the evaluation of the different regimens were:

- Number of relapses. Relapse was defined as the reappearance of signs or symptoms of the disease or positive culture results after completion of therapy during follow-up, all occurring after an asymptomatic period.
- Therapeutic failure, defined as persistence of signs and/or symptoms beyond a period after the beginning of treatment considered appropriate in the various studies.
- A combined variable comprising relapse and therapeutic failure taken together.
- Side effects of the various regimens, classified as serious when withdrawal of the drug was required, and as moderate when treatment withdrawal or change of therapy was not required.
- Mortality.

Another aim was to determine the time lapse between the start of antimicrobial treatment and the disappearance of fever and other symptoms.

### Data extraction and quality assessment

Two investigators extracted independently data from studies. Discrepancies were noted and discussed between reviewers and resolved by consensus. For each study, the following data were recorded:

- Year of publication
- Study type (randomization and blinding)
- Number of patients treated.
- Diagnostic criteria and exclusion criteria.
- Number of patients lost to follow-up and the reason for it.
- Antibiotic regimens used, with dosage and duration, as well as route of administration
- Percentage of patients with focal disease in each study
- Number of relapses and treatment failures
- Time lapse between the beginning of treatment and the disappearance of fever or symptoms
- Follow-up period
- Side effects of medication, with indication of effects requiring treatment withdrawal
- Mortality
- Whether patients were admitted to a hospital at the beginning of or during treatment.

The adequate generation of allocation sequence and concealment of allocation (assessment of selection bias), blinding (assessment of performance bias), and incomplete outcome data (attrition bias) was determined for each study. These components were graded as high risk, low risk or unclear risk of bias (table S3).

### Data synthesis and analysis

For comparative randomized clinical trials, an analysis was performed comparing the various therapeutic regimens in terms of relapse, therapeutic failure, the combined variable of relapse and therapeutic failure, mortality and side effects, wherever such comparison was possible. The differences between the two regimens compared in each case are expressed as an odds ratio with the relevant confidence interval (CI95%), and were contrasted using the Mantel-Haenszel test, using the procedure for random effect and stratified analysis where heterogeneity between studies was found. Where the use of these methods was necessary, it is indicated in the text. The Rosenthal tolerance index and a funnel plot were used to evaluate potential selection bias in the studies. Cochran's Q statistic and the I^2^ inconsistency statistic were used to measure heterogeneity regarding study results. In all statistical tests, the level of statistical significance used was p<0.05. Analyses were performed using RevMan version 5.

## Results

Details of the clinical trials on the treatment of brucellosis are given in Table S4. Table S5 shows non-randomized and non-comparative studies designed to assess a given treatment regimen.

### Description of the different regimens studied

#### Combinations involving parenteral drugs

A combination of tetracyclines and streptomycin is the most widely studied of the regimens including aminoglycosides. In a study published in 1985, Ariza *et al*. [8] investigated a regimen comprising 3 weeks of streptomycin and 30 days of oral tetracycline or doxycycline, used indiscriminately, in 28 patients. Acocella *et al*. [10] investigated a shorter regimen comprising 2 weeks of streptomycin and 21 days of tetracycline. There is a wide discrepancy between the results of these two studies: in the first, relapse occurred in only 7.1% of patients compared to 22.2% in the latter, to which an 18.52% treatment failure rate must be added, including one patient who stopped taking the medication due to side effects.

Twelve studies investigated doxycycline and streptomycin with similar results [10], [11], [17], [18], [20], [24]–[30]. One of these studies was performed in children and the study by Cisneros *et al.* [24] was non-comparative. Considering the remaining 10 studies, the regimen of doxycycline and streptomycin was investigated in 597 patients. Twenty seven patients had a relapse (4.5%; range: 0–9.7%), and the percentage of relapse was less than 10% in any of the studies. The combined variable of relapse and treatment failure is 44 patients (7.4%: range: 0 and 12.5%). The study with the highest rate of relapse and treatment failures combined was that by Ersoy *et al.* published in 2005, with 12.8% [26]. One group of 4 studies used streptomycin for 3 weeks [11], [25], [26], [29] and a second group of 6 studies used streptomycin for 2 weeks [10], [17], [18], [20], [27], [28]. The overall percentage of relapse and treatment failure in the former was 6.9% (10 out of 145 patients), while in the latter was 7.5% (34 out of a total 452 patients) (p = 0.96). Eight of these 10 studies involved comparison with doxycycline-rifampicin [10], [11], [17], [18], [20], [25], [26], [29], two compared this regimen with doxycycline and gentamicin [27], [28] and one included a comparison with rifampicin and ofloxacin [26]. In all these studies doxycycline-streptomycin combination was superior or equal than the other compared regimens. The average time until the disappearance of fever in all these 10 studies was less than a week.

There are only 4 studies on the combined use of doxycycline and gentamicin in adults. Hasanjani *et al*. published the results of two comparative studies of doxycycline and gentamicin with doxycycline and streptomycin [27], [28]. In the first one, doxycycline for 45 days plus gentamicin for 7 days of was compared with doxycycline for 45 days plus streptomycin for 14 days. In the second study published in 2010 [28], doxycycline treatment was extended to 8 weeks and combined with 5 days of gentamicin, and this treatment was again compared with doxycycline for 45 days plus streptomycin for 14 days. In none of the studies was any advantage noted for the combinations including gentamicin instead of streptomycin.

Solera *et al* performed 2 studies [31], [32] comparing doxycycline for 30 or 45 days with fixed treatment duration of 7 days for gentamicin. The first study was non-randomized and included fewer patients. The second study, published in 2004, was a randomized double-blind study and included not only a comparison of the two treatment durations but also an analysis of relapse risk factors. The results of the two studies were in favor of the administration of doxycycline for 45 days. In the study of relapse risk factors, the significant factor was in fact the shorter treatment time.

Although in a non-comparative study, other combination including aminoglycosides that has been tested is doxycycline plus netilmicin [33]. This regimen resulted in a high rate of relapse (12.5%) and therapeutic failure (7.7%).

There has only been one small non-comparative study in 10 patients [34] to investigate azithromycin instead of doxycycline for 21 days in combination with gentamicin for the first 7 days. Three out of 10 patients experienced relapse, treatment failed in 2, and treatment had to be withdrawn in the case of one patient due to side effects.

Only one study investigated triple therapy with doxycycline, rifampicin and amikacin [16] in patients with uncomplicated brucellosis, with the aminoglycoside being administered for 1 week and the other two antimicrobials for a total of 8 to 12 weeks. Comparison was made with the regimen of doxycycline and rifampicin administered together for 8 to 12 weeks. No significant differences between the two combinations were observed regarding relapse (6 cases, i.e. 5.7%, of relapse with triple therapy vs. 9 cases, i.e. 9.3%, with combined doxycycline-rifampicin; p = 0.4). However, differences were observed in terms of resolution of symptoms (fever, arthralgia, shivering) but with only borderline statistical significance (p = 0.04, CI 95%: 0.008–0.15). This difference is more pronounced when fever is taken as a symptom (although the study does not specify what temperature constitutes fever) and comparisons of number of patients with fever 2 weeks after initiating treatment, with 32% of patients presenting fever after two weeks with doxycycline and rifampicin vs. 7.8% of patients on the triple therapy. In addition, there is a discrepancy between the time to defervescence described in the text and that shown in the graph published in the study, which suggests that over 50% of patients presented fever after two weeks of treatment. This high percentage of patients with fever after two weeks of treatment was not seen in any of the other studies investigating combined doxycycline and rifampicin [8], [17]–[22].

Triple therapy was administered in two other studies [15]–[25], however, they involved only patients presenting osteoarticular brucellosis. Bayindir *et al.* [15] carried out a study in patients with brucella spondylitis. Twenty two patients receiving combined rifampicin and doxycycline for 45 days, together with streptomycin over the first 15 days were included in the study and the results were extremely good regarding relapse and treatment failure, with a 100% response rate and no relapses. In the second study, conducted in Egypt [35] in patients presenting osteoarticular brucellosis, dual therapy comprising rifampicin and either co-trimoxazole or doxycycline was compared with triple therapy comprising doxycycline, rifampicin and streptomycin. Although the results of the last combination were superior, multivariate analysis showed that treatment duration (less than 5 months) was the variable truly predictive of relapse. Despite this and the limitations of the study in terms of treatment allocation, the authors recommend triple therapy in the conclusion to their article.

#### Combinations of orally-administered drugs

The regimen combining rifampicin and tetracycline is the most common in clinical trials of human brucellosis, with 20 studies investigating this type of regimen [8], [10], [11], [16]–[22], [25], [26], [29], [30], [36]–[41]. Nine of these studies compared this regimen with one of tetracycline plus streptomycin, and 6 studies [19], [21], [22], [26], [37], [38] compared the combination of rifampicin with a quinolone. Two studies compared this regimen with doxycycline and a quinolone [36], [38]. One of these studies compared it with monotherapy involving ciprofloxacin, although in less than 10 patients per regimen [41]. Another study involved minocycline instead of doxycycline but this was a retrospective study [40]. The study by Lubani *et al.* [30] was conducted only in children. Alavi *et al.* [39] compared this regimen with combined doxycycline and trimethoprim/sulfamethoxazole. Finally, the only study comparing doxycycline and rifampicin *vs.* triple therapy with rifampicin, doxycycline and amikacin is the previously indicated work of Ranjbar *et al.* [16]

In general, less satisfactory results were obtained in comparison with combined streptomycin and doxycycline, with relapse and treatment failure rates of over 20% in certain studies, although the results of the various studies were highly disparate. Relapse rates ranged from 38.8% described by Ariza *et al.* [8] with administration of this combination for 30 days to 3.3% reported by Akova *et al.* [19] following administration of the regimen for 6 weeks. However, other authors using this combination for 45 days reported a higher relapse rate than Akova, with Solera *et al.* [20] indicating a 16% relapse rate and an 8% treatment failure rate. Time to defervescence with this regimen was less than a week in almost all cases (Table S1), and it was the best-tolerated regimen, with treatment withdrawal for adverse drug effects being extremely rare.

Only three studies investigated combined co-trimoxazole and tetracyclines, two in adults [42], [39] and one in children [30]. The study by Hasanjani *et al.* [42] reported better results for tetracycline and co-trimoxazole (8.6% relapse and 7.1% treatment failure) than with rifampicin and co-trimoxazole (p = 0.646 for relapse, p = 0.02 for treatment failure, and p = 0.028 for the combined variable of relapse and treatment failure). Alavi *et al* [39] compared combined doxycycline and co-trimoxazole with combined doxycycline and rifampicin in a population of nomadic patients in Iran. The relapse and treatment failure rates with doxycycline and co-trimoxazole were 5.88% and 1.94% respectively. With doxycycline and rifampicin, the corresponding rates were 11.76% and 9.81% respectively. The difference with regard to the combined variable for relapse and treatment failure obtained was p = 0.05. Finally, Lubani *et al.* [30] obtained excellent results with this combination in their study in children, with an overall relapse rate of 4.9%, and no treatment failures for therapy lasting 8 weeks.

Only three studies were performed with combined co-trimoxazole and rifampicin: two in children [30], [43], and one in adults [42]. In 2004, Hasanjani *et al.* [42] published a study in 280 patients aged 10 and over (range: 10 to 81 years) comparing co-trimoxazole and rifampicin *vs.* co-trimoxazole and doxycycline (140 patients in each group). Co-trimoxazole and rifampicin were administered for two months with a high rate of treatment failure (16.4% failures, 10% relapses). In 2006, the same author investigated this regimen [43] in 130 children divided in two groups, one treated for 6 weeks and the other for 8 weeks. The results were superior to those in the previous study and the combined variable of relapse and treatment failure rate was lower in the 8-week group (4.5%). Such good results in children had been previously reported by Lubani [30] in 1989. In this study 34 children received this regimen with a global relapse rate of 5.88%, but it should be noted that patients experiencing relapse had received treatment for less than 6 weeks.

Six studies [19], [21], [22], [26], [37], [38] combined quinolone and rifampicin. In two [21], [38] the quinolone was ciprofloxacin, and in both it was compared to doxycycline and rifampicin, with the quinolone yielding the least satisfactory results. In the remaining four studies [19], [22], [26], [37], the quinolone used was ofloxacin. Results were disparate, although superior to those obtained with ciprofloxacin, and with a relapse rate of approximately 10%. In both studies comparison was made with combined doxycycline and rifampicin. In the remaining studies considered separately, with the exception of a study by Akova *et al.* [19], the results for ofloxacin and rifampicin as regards relapse and treatment failure rates were similar to those obtained with doxycycline and rifampicin.

Two studies investigated this regimen in patients presenting brucellar spondylitis. Bayindir *et al.* [15] administered ofloxacin and rifampicin for 45 days to 19 patients with spondylitis, with poor results, comprising a treatment failure rate of 26% and a 26% relapse rate. Alp et *al.* [44], administered ciprofloxacin and rifampicin for a minimum of 12 weeks, with better results. A 100% response rate was achieved with no relapses, although the authors reported 2 and 9 patients having moderate and mild sequelae, respectively.

Only two comparative studies assessed quinolone-doxycycline combination [36], [38], and in both the quinolone was ciprofloxacin. In the first study [36] in of 12 patients, only one patient relapsed (8.33%) with no treatment failures. In the second [38], the results were inferior to those with doxycycline and rifampicin or with ciprofloxacin and rifampicin, with a relapse rate of 17.5% *vs.* 7.7% and 8.3% respectively, but with p = 0.35. However, the data for the doxycycline-quinolones treatment are very scarce and its inferiority versus quinolone-rifampin is not evident.

#### Monotherapy

Very few comparative studies were found that assess of monotherapy [9], [25], [30], [41], [45] and they included only a small number of patients.

In all the studies with co-trimoxazole, high rates of treatment failure and relapse [9], [25], [30] were recorded. Ariza *et al.* [9] carried out a study comparing monotherapy with co-trimoxazole for 45 days vs. combined tetracycline for 21 days and streptomycin for the first 14 days. The relapse rate was 46.6% in the co-trimoxazole group vs. 14.8% in the tetracycline-streptomycin group (p<0.05).

In the study by Montejo *et al.* [25], 64 patients received co-trimoxazole for 6 months, with an 81.25% cure rate. In over 18% of the patients a change of treatment was required due to side effects (3.1%), treatment failure or non-completion (12%) and relapse (3.1%).

The study by Lubani *et al.* [30] included 161 children treated with co-trimoxazole, but the relapse rate was 29.8% no matter whether the, treatment period was 3, 5 or 8 weeks.

The three studies performed with tetracyclines were those by Feiz *et al.* [46], Lubani *et al.* [30] and Montejo *et al.* [25]. That of Feiz [46] in Iran was published in 1973 and it thus lies outside the timeframe of our study. Nevertheless Feiz reported a 31% relapse rate in patients treated with oxytetracycline and a 29% relapse rate in those treated with doxycycline, although the treatment period was very short, lasting only 21 days. Moreover, the dose was reduced to half the initial dose 14 days after the beginning of treatment in the doxycycline group. These two circumstances make difficult to compare this work with subsequent studies.

As indicated above, the study by Lubani *et al.* [30] was conducted in children, and also tested two regimens, one with oxytetracycline and the other with doxycycline. The results were far superior to those reported by Feiz, and relapse rates proved to be inversely proportional to treatment time, a finding that was much clearer with this regimen than with others used in the same study. Thus, the relapse rate dropped to 0% when the treatment was extended to 8 weeks.

The third study published was that of Montejo *et al.* [25] in 1993. For the purpose of the study, a daily dose of 200 mg doxycycline was administered constantly during the six weeks of treatment. Seventy-one patients were included, with a relapse rate of 14.08%. Since then, no other studies on monotherapy with tetracyclines have been published.

The results of the study published in 1987 by Grasso *et al.* [47] in Italy are also worth commenting, even though it is a retrospective non-comparative study. It was performed in 295 patients with brucellosis treated with minocycline. While 116 patients received a single course for 35 to 40 days, 82 others received two courses of minocycline lasting 35 and 15 days with a 15-day pause between cycles, and the remaining 97 patients received it with a vaccine. In all cases, the dose was 100 mg/12 hours. The overall number of patients presenting relapse was 10 (3.38%). One patient died due to hepatic failure not clearly related to the treatment received in the study. The authors compared these results with those obtained in another sample of 179 patients treated with doxycycline in which the relapse rate was 5.17%. However, this study exhibits the limitations of a retrospective study, and furthermore, no clear details are offered of total patient follow-up time.

The only prospective, comparative and randomized study with quinolone monotherapy was published in 1990 by Lang *et al.* [41]. This was a small study in 6 patients with brucellosis treated with ciprofloxacin for 42 days. The comparison was established with 4 other patients treated with doxycycline and rifampicin. Relapse occurred in 5 of the 6 patients treated with ciprofloxacin.

Subsequently, no comparative randomized studies of quinolones as monotherapy have been published. In 1991, Khuri-Bulos and Shaker [48] published a small study in 5 patients each receiving 200 mg ofloxacin every 12 hours for 21 days. Relapse occurred in 3 of the patients. Doganay and Aygen [49] published a small non-comparative study in 1992 in 14 patients receiving 500 mg ciprofloxacin every 12 hours for 3 to 6 weeks. Three exhibited relapse (21.4%) and one patient presented endocarditis and died 5 months later. Al Sibai *et al.* [50] carried out another non-randomized prospective study in 1992 in 16 patients treated with ciprofloxacin for 6 to 12 weeks, with a relapse rate of 27%.

Rifampicin was used as monotherapy in several studies. The largest of these was the already-mentioned study by Lubani *et al.* [30], with positive results in children. Few other studies have been published [51] and only in small numbers of patients. The risk of inducing resistance of tuberculosis to rifampicin is likely to have curbed the implementation of such studies.

Two studies were performed with ceftriaxone [45], [52] in a small number of patients and with poor results. That of Lang [45] was a small comparative study in only 8 patients receiving ceftriaxone, and the treatment failure rate was 75% (6 patients). Al Idrissi [52] carried out a non-comparative study, also with a very high treatment failure rate (30.8%).

### Comparisons between regimens used in randomized clinical trials

The comparisons of regimens that can be established with the randomized clinical trials published in the past 25 years are as follows: doxycycline and streptomycin *vs.* doxycycline and rifampicin, 9 studies; doxycycline and streptomycin *vs.* doxycycline and gentamicin, 2 studies: doxycycline and rifampicin *vs.* quinolone and rifampicin, 6 studies; doxycycline and rifampicin *vs.* ofloxacin and rifampicin, 4 studies; and doxycycline and rifampicin *vs.* doxycycline and quinolone, 2 studies. Since there was only one study [16] using doxycycline, rifampicin and aminoglycosides vs. other regimens in patients with uncomplicated brucellosis, no conclusions can be drawn on the value of this triple therapy. Other studies using this triple therapy were performed only in patients with brucellosis involving osteoarticular complications and, since the importance of these regimens might be overestimated, it seems unsuitable to extrapolate such data to patients with acute non-focal brucellosis. In addition, the only monotherapy on which more than one randomized study was performed was with co-trimoxazole, and the results show a high relapse rate [9], [25]. The only comparative randomized study with tetracyclines in adult patients in monotherapy published in the past 25 years is that of Montejo [25]. The study by Feiz [46], besides being outside the timeframe of our review, is not comparable with the study by Montejo in terms of either dosage or treatment duration.

The study by Acocella *et al.* [10], which is important for the comparison of doxycycline-streptomycin vs. doxycycline-rifampicin, included 3 groups. One group was treated with tetracycline for 21 days and streptomycin for 14 days. This regimen is not comparable with others using doxycycline 45 days because of the shorter tetracycline treatment. Thus, the comparison of doxycycline-streptomycin *vs.* doxycycline-rifampicin included only the other two groups in the study, which received these two regimens.

**Figure 2:**
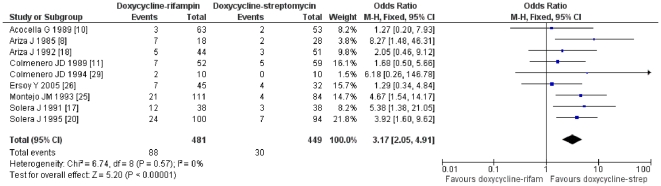
Relapses and treatment failure with doxycycline and rifampicin vs. doxycycline and streptomycin regimens in treatment of human brucellosis.

The results of the comparison between doxycycline-streptomycin and doxycycline-rifampicin favor the first combination in terms of relapse (OR = 3.52; CI95% = 2.14–5.81; p<0.001;Rosenthal index 57; I^2^ = 0%) and of the combined variable of relapse and therapeutic failure (OR = 3.17; CI95% = 2.05–4.91; p<0.001; Rosenthal index = 64; I^2^ = 0%) (figure 2 and figure S1).

The difference in the comparison between doxycycline-streptomycin and doxycycline-gentamicin is not statistically significant as regards either relapses (OR = 1.65; CI95% = 0.53–5.15; p = 0.38621; I^2^ = 0%), or the combined variable of relapse and therapeutic failure (OR = 1.89; CI95% = 0.81–4.39; p = 0.14106; I^2^ = 0%).

**Figure 3:**
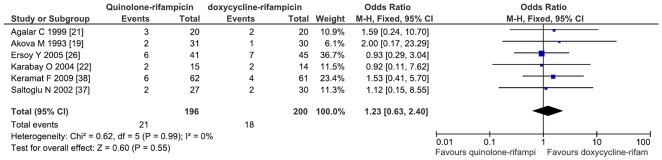
Relapses and treatment failure with quinolone-rifampicin regimen versus doxycycline and rifampicin regimen in treatment of human brucellosis.

**Figure 4:**
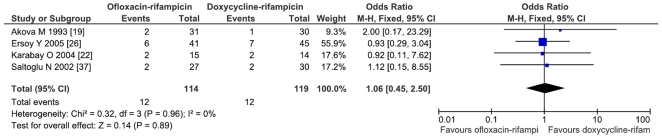
Relapses and treatment failure with ofloxacin and rifampicin versus doxycycline and rifampicin in treatment of human brucellosis.

The comparison of doxycycline-rifampicin vs. rifampicin-quinolone shows no significant differences neither for relapse (OR = 1.11; CI95% = 0.53–2.34; p = 0.77) nor for the combined variable of relapse and treatment failure (OR = 1.23; CI95% = 0.63–2.40; p = 0.55; I^2^ = 0%) (figure 3). The comparison of the doxycycline-rifampicin regimen with the four studies involving ofloxacin-rifampicin shows no differences (for relapse and treatment failure: OR = 1.06; CI95% = 0.45–2.5) (figure 4).

Finally, the comparison of doxycycline-quinolone *vs.* doxycycline-rifampicin favors doxycycline-rifampicin (OR = 3.92; CI95% = 1.35–11.42; p = 0.01). When comparing combined regimens containing quinolones (whether doxycycline-quinolone or rifampicin-quinolones) with combined doxycycline-rifampicin, the OR = 1.33 for relapse was not statistically significant (CI95% = 0.67–2.63; p = 0.42; I^2^ = 0%). As regards the combined variable of relapse and treatment failure, the result was similar (OR = 1.59; CI95% = 0.87–2.91; p = 0.14; I^2^ = 0%).

### Side effects

The side effects of the standard medication were mild or moderate, and only rarely serious and requiring treatment withdrawal. Not all authors reported side effects, and these were not uniformly evaluated in all cases. The numbers of side effects reported are shown in Table 1.

**Table 1:** Side effects reported in different studies in the treatment of non-focal human brucellosis.

Regimen	N° of studies [references]	N° patients	Light to moderate side effects (%)	Serious side effects (%)*
Tetracycline + streptomycin	2 [8], [10]	55	8 (14.5%)	1 (1.8%)
Doxycycline + streptomycin (15 days)	8 [10], [17], [18], [20], [24], [25], [27], [28]	591	98/551 (17.8%)	5 (0.8%)
Doxycycline + streptomycin (21 days)	3 [11], [25], [26]	135	15/91 (16.5%)	0
Doxycycline (30 days)+ gentamicin	2 [31], [32]	108	43 (39.8%)	0
Doxycycline (45 days)+ gentamicin	3 [27], [31], [32]	187	60 (32.1%)	0
Doxycycline (≥56 days)+ gentamicin	1 [28]	82	25 (30.5%)	0
Triple therapy	1 [16]	110	6 (5.5%)	0
Doxycycline + rifampicin (28–30 days)	2 [8], [25]	83	1/18 (5.6%)	0
Doxycycline + rifampicin (42–45 days)	12 [10], [11], [17]–[22], [25], [26], [36], [37]	482	103/406 (25.4%)	6 (1.2%)
Doxycycline + rifampicin (≥56 days)	3 [16], [38], [39]	224	14/171 (8.2%)	2 (0.9%)
Rifampicin + cotrimoxazole	1 [42]	140	----------	7 (5%)
Tetracycline + cotrimoxazole	1 [42]	140	----------	2 (1.4%)
Rifampicin + ciprofloxacin	2 [21], [38]	82	4 (4.9%)	0
Rifampicin + ofloxacin	4 [19], [22], [26], [37]	114	14/87 (16.1%)	1(0.9%)
Doxycycline + quinolone	1 [38]	55	9 (16.4%)	0
Cotrimoxazole	1 [25]	64	----------	2 (3.1%)
Doxycycline	1 [25]	71	----------	1 (1.4%)
Quinolones	1 [50]	16	7 (43.7%)	0

**Figure 5:**
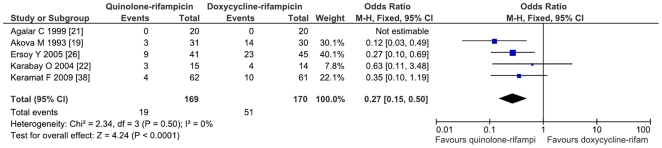
Side effects with combined rifampicin and quinolone versus combined doxycycline and rifampicin in patients with human brucellosis.

Where comparison was possible using data from the separate randomized comparative studies, the only differences concerning side effects were noted in the comparison of doxycycline-rifampicin vs. rifampicin-quinolones, with the result in favor of the latter combination (OR = 0.27; CI95% = 0.15–0.50; p<0.0001; I^2^ = 0%; Rosenthal index = 18) (Figure 5).

Seven of the studies that compared between doxycycline-streptomycin and doxycycline-rifampicin considered side effects. In this respect, the doxycycline-streptomycin regimen has not advantage, whether we consider the side effects in general (OR = 1.13; CI95% = 0.58–2.18; p = 0.73; I^2^ = 63% p = 0.01; random effects method) or the serious side effects (OR = 1.52; CI95% = 0.44–5.29; p = 0.51). Analysis of side effects in general showed heterogeneity among studies. This heterogeneity is due to the presence of two groups of studies in which side effects were considered. In a group of four studies [8], [10], [11], [17], the comparison favours doxycycline and rifampicin (OR = 0.52; CI95% = 0.27–0.99; p = 0.05) while a second group of three studies [18], [20], [26] favors the doxycycline-streptomycin combination (OR = 2.08; CI95% = 1.32–3.27; p = 0.002).

There were no significant differences in side effects between doxycycline-streptomycin and doxycycline-gentamicin (OR = 0.74; CI95% = 0.46–1.19; p = 0.22).

### Treatment duration

The relapse and treatment failure rates by regimen and by treatment duration are shown in Table 2.

**Table 2:** Percentage, by regimen and treatment duration, of relapses and therapeutic failures reported in different studies in treatment of non-focal human brucellosis.

Regimen	N° of studies	patients	relapses	failures	Relapses + failures
TETR (21 days)+STP	2 [46], [10]	55	16.4%	9.1%	25.5%
TETR (30 days) + STP (21 days)	1 [8]	28	7.1%	0%	7.1%
DX(45 d)+STP(15 d)	8 [10], [17], [18], [20], [24], [25], [27], [28]	591	4.2%	3.4%	7.6%
DX (45 d) +STP(21 d)	3 [11], [25], [26]	135	5.2%	1.5%	6.7%
DX (30 days) + G	2 [31], [32]	108	21.3%	0%	21.3%
DX (45 days) + G	3 [31], [32], [27]	187	6.9%	1.1%	8.02%
DX (56 days or more) +G	1 [28]	82	2.4%	2.4%	4.9%
DX + RF (28–30 days)	2 [8], [25]	83	25.3%	1.2%	26.5%
DX+ RF (42–45 days)	12 [10], [11], [17]–[22], [25], [26], [36], [37]	494	12.1%	3%	15.2%
DX+RF (56 days)	3 [16], [38], [39]	222	7.7%	9%	16.6%
RF + OFX (30 days)	1 [22]	15	13.3%	0%	13.3%
RF+ OFX (42–45 days)	3 [19], [26], [37]	99	8.1%	2%	10.1%
DX + TMP/SMX (adults)	2 [42], [39]	191	7.9%	5.8%	13.6%
RF + TMP/SMX (adults)	1 [42]	140	10%	16.4%	26.4%
DX (adults) 21 days	1 [46]	31	29%	0%	29%
DX (adults) 42 days	1 [25]	71	14.08%	0%	14.08%

Abbreviations: DX = doxycycline; RF = rifampicin; TETR = tetracycline u oxitetracycline; STP = streptomycin; G = gentamicin; TMP/SMX = cotrimoxazole; OFX = ofloxacin.

All trials involving doxycycline and streptomycin involved a treatment duration of six weeks. The only difference here concerns the duration of treatment with streptomycin, which in two publications [25], [26] was three weeks rather than the standard two weeks. However, this prolonged parenteral treatment was not clearly beneficial in terms of results.

**Figure 6:**
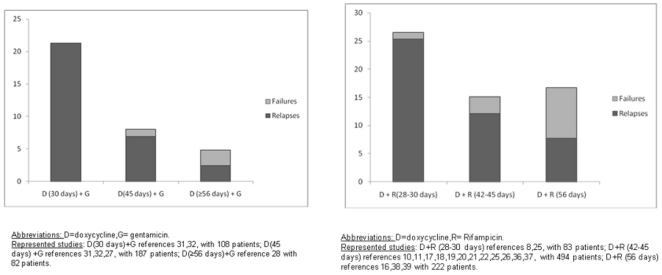
Relapses and therapeutic failures with the combined doxycycline-gentamicin regimen and doxycycline-rifampicin regimen reported by treatment duration.

Regarding combined doxycycline and gentamicin, it may be concluded that treatment with doxycycline for 45 days yields superior results to 30 days of treatment [31], [32]. Abramson *et al.* [53] investigated a short course of treatment in 10 children aged between 8 and 16 with gentamicin for 5 days and doxycycline for 3 weeks, with a high rate of relapse and treatment failure (3 patients: 30%). In contrast, in a recent study [28], treatment was maintained for 8 weeks with doxycycline and gentamicin, but no clear advantages were observed compared with 45 days of treatment (figure 6).

Similar results were found for the doxycycline-rifampicin regimen (figure 6). The majority of authors continued antibiotic therapy for 45 days. Only two [8], [25] shortened the treatment to one month, with clearly inferior results (Table 2). Solera *et al.* reduced the treatment time with rifampicin to 21 days, maintaining doxycycline for 45 days, but this also yielded poor results [17].

In the studies combining rifampicin and quinolone, a 30-day treatment duration yielded a higher relapse rate than a 45-day treatment duration, no matter whether the quinolone was ciprofloxacin (8.3% relapses in Keramat *et al.* [38], with 8 to 12 weeks of treatment vs. 15% in Agalar *et al.* [21] with 30 days of treatment) or ofloxacin (the highest relapse rate with this quinolone was seen in Karabay *et al.* [22]: 13.3% for 30 days of treatment).

The results obtained by Feiz [46] following 21 days of treatment with tetracyclines show a very high relapse rate, but these results were better after 45 days of treatment in the study by Montejo *et al.* [25]. Lubani *et al.* [30] showed that the results shows no significant differences dependent on treatment time in children.

There were only two comparative randomized studies comparing similar regimens but with different treatment durations. One of these studies is that by Acocella *et al.* [10], comparing doxycycline for 45 days and streptomycin for 14 days vs. tetracycline for 21 days and streptomycin for 14 days. Montejo *et al.* [25] administered streptomycin for 14 or 21 days with 45 days of doxycycline, and doxycycline and rifampicin for 4 or 6 weeks. Comparisons cannot be drawn combining data from the different studies.

### Dose

The dosage of drugs used was fairly uniform throughout the various studies. For doxycycline, streptomycin and gentamicin, the dose used was similar in all trials. The only study that used two distinct doses of the same drug sequentially was that of Feiz [46], in which separate doses were used in monotherapy with oxytetracycline and with doxycycline. The high relapse rate observed in this study could be due to either this change of dosage or to the brief duration of treatment (only 21 days). For rifampicin, doses of 600 and 900 mg per day (in certain studies a dose of 15 mg/kg bodyweight was stipulated) were used in combination with doxycycline, but no clear differences were observed between the results of the different studies.

### Mortality

The data provided by the various studies, whether including trials or series of clinical case reports (Table 3), show that the mortality associated with brucellosis is very low. No deaths were reported in the randomized studies, and in the case reports which we examined, only 6 deaths were reported for over 1500 cases of brucellosis (<0.4%). However, in some studies, conducted primarily in developing countries, initial treatment involved hospitalization (Tables S1, S2 and 3).

**Table 3:** Results of brucellosis therapy in large patient series.

Author (year) [ref]	country	Years of study	N° cases	Therapeutic regimens	relapses	Failures	mortality	Hospital admission
**Barroso Garcia P (2002)** [56]	Spain	1972–1998	1595	TETR+STP ; TETR+STP+ Sulphonamide ; DX+STP ; STP+Sulphonamide+DX ; TETR	NR	NR	NR	No
**Andropoulos (2007)** [59]	Greece	1990–2003	144	DX+STP (>14 años); RF+TMP/SMX (niños)	4 (3%)	NR	0	Yes
**Bosilkovski (2007)** [57]	Macedonia	1998–2004	418	DX+RF+TMP/SMX ; DX+RF ; DX+RF+G	16.2%	10.4%	1	Yes (until improvement)
**Aygen (2002)** [55]	Turkey	1989–1998	480	DX+RF; DX+STP; TETR+STP; CPX	26 (5.4%)	0	3	NR
**Memish (2000)** [58]	Saudi Arabia	1983–1995	160	TETR+STP; DX+RF; TETR+STP+ RF; RF+TMP/SMX; RF+STP+TMP/SMX	7	NR	0	Yes
**Buzgan (2010)** [65]	Turkey	1998–2007	1028	DX+RF (most used); DX+STP; DX+RF+STP; DX+CPX; RF+TMP/SMX; RF+CPX	4.7%	NR	NR	Yes (2–3 weeks)
**Al Shaalan (2002)** [60]	Saudi Arabia	1984–1995	115 (children)	RF+TMP/SMX+STP; STP+TMP/SMX; RF+TMP/SMX; STP+TETR+TMP/SMXl; RF+DX+STP	8	NR	1	Yes
**Savas l (2007)** [61]	Turkey	2000–2002	140	DX+RF; DX+STP; RF+CPX; DX+RF+STP; RF+TMP/SMX	5	NR	0	Yes(37 patients)
**Demirturk (2008)** [62]	Turkey	2002–2006	99	DX+RF; DX+RF+CPX	0 (of 30 cases with follow up)	0	1	Yes

TETR = tetracycline, STP = streptomycin, DX = doxycycline, RF = rifampicin, TMP/SMX = cotrimoxazole, G = gentamicin, CPX = ciprofloxacin.

### Time to defervescence

Another data item used in the studies is time to defervescence. Although this time varied in all publications, it was under a week in practically all cases, and in most less than 5 days (Tables S4 and S5). Malik [54] published a study that included a retrospective analysis of 73 patients diagnosed with brucellosis in a hospital in Saudi Arabia between 1987 and 1994. The mean defervescence time was 4.32±1.47 days, with no differences between treatment groups. Data were also provided concerning the duration of the hospital stay, which was 7.75±2.12 days. In the study of Aygen *et al.* [55], among the 187 patients with fever, the average time to defervencence for all treatments regimens was less than 7 days (range: 2 to 15 days).

### Results in large patient series

Studies including over 100 patients published in the past 10 years are shown in Table 3. These studies include regimens for which there are no studies demonstrating efficacy. Thus, Barroso *et al.* [56] report the use of triple therapy comprising tetracycline, streptomycin and a sulphonamide. Bosilkovski *et al.* [57] report the use of doxycycline with rifampicin and gentamicin. Memish *et al.* [58] report the use of combined tetracycline, rifampicin and streptomycin.

The study by Aygen *et al.* [55] of 480 cases is significant, since it shows the high relapse rate in patients receiving ciprofloxacin, whether as monotherapy or in combination. Other studies [59]–[62] are included in table 3.

In these series of patients, fewer cases of relapse were observed than in randomized clinical trials, probably as a result of the closer follow-up of patients included in clinical trials.

## Discussion

Even though effective antibiotics for brucellosis are available, the problem of treating this disease has not been completely solved. The most widely used and recommended regimens are those combining doxycycline and an aminoglycoside or rifampici n. Other regimens that have demonstrated efficacy in different studies are combinations of quinolones and rifampicin, co-trimoxazole and rifampicin, and triple regimens with doxycycline, rifampicin and aminoglycoside. In addition, observational studies in large series of patients [55]–[57] show that regimens other than those recommended continue to be used like, for example, doxycycline, rifampicin and co-trimoxazole, rifampicin and ciprofloxacin, doxycycline and ciprofloxacin, or regimens based on antibiotics used in monotherapy [58].

Therefore, finding a unique response in the treatment of brucellosis is not an easy task. In our review, we concentrated on acute non-focal brucellosis. Our intention in this study was to investigate, through a review of published data, the possibilities of different regimens, representing an alternative to those recommended most frequently. With these data we sought to answer the most important questions concerning the treatment of human brucellosis and which we posed at the end of the introduction: what is the most effective regimen, what alternative regimens are there, whether triple therapy may be recommended as the best option, whether monotherapy represents a valid alternative, and what treatment duration should be recommended. We also sought to identify new fields of research in view of current knowledge and the complexity of this disease.

Based on the studies performed to date, we may conclude that the regimen combining doxycycline and streptomycin has been shown to be superior to combined doxycycline and rifampicin, in terms of both relapse rate (OR = 3.52; CI95% = 2.14–5.81) and combined relapse-treatment failure (OR = 3.17; CI95% = 2.05–4.91). No advantages emerged regarding side effects in view of the heterogeneity of the various studies in this respect. No significant differences were obtained with combined doxycycline and gentamicin to combined doxycycline and streptomycin, which has been the most widely-used aminoglycoside.

In spite of these findings, the need for parenteral administration of aminoglycosides may complicate the use of this regimen, primarily in those parts of the world where lack of healthcare personnel is a limiting factor [63]. In a recent study [64], 64.6% of healthcare professionals interviewed preferred the doxycycline-rifampicin regimen, despite the demonstrated superiority of streptomycin-doxycycline. Furthermore, in a recent large series involving over 1000 patients [65], the most frequently-used regimen was doxycycline-rifampicin. Indeed,combined rifampicin and doxycycline also poses problems in developing countries due to its potential to induce resistance to rifampicin in other infections, mainly in tuberculosis.

The only study involving triple therapy in patients with acute uncomplicated brucellosis is that of Ranjbar [16], which investigated combined doxycycline, rifampicin and amikacin. Amikacin was administered parenterally for one week, and the other two antibiotics for 8–12 weeks. This regimen, which is longer than the standard treatment, is potentially disadvantageous in terms of therapeutic adherence and cost. Furthermore, a comparison was made with combined doxycycline-rifampicin, and no clear differences were observed concerning therapeutic efficacy. The conclusions were based on the disappearance of fever after two weeks of treatment, a time extremely long for evaluating the clinical response which, as already stated, occurred in all studies within the first few days of the start of treatment [56], [66]. Other studies of triple therapy were performed in patients presenting osteoarticular brucellosis [15], [35], and therefore the conclusions of these studies cannot be extrapolated to patients with uncomplicated brucellosis. As a result, this triple therapy cannot be recommended as the treatment of choice for human brucellosis until data supporting its usefulness is obtained in randomized clinical trials.

Combined rifampicin and quinolone appears to offer a valid alternative, with efficacy comparable to that of combined doxycycline and rifampicin in terms of both relapse (OR = 1.11; CI95% = 0.53–2.34) and combined relapse-treatment failure (OR = 1.23; CI95% = 0.63–2.40). In addition, comparison of side effects favors the regimen with quinolone (OR = 0.27; CI95% = 0.15–0.50). However, no statistical significance was noted when comparing combined doxycycline-quinolone with doxycycline-rifampicin. However, most studies with quinolones have been made in the same group of countries and some of them include a small number of patients. Consequently, although the results obtained indicate that quinolones could be an alternative to doxycycline plus rifampicin, the recommendation of its use may require further studies.

Skalsky *et al.* [14] advise against the use of regimens involving quinolones. However, the results of their meta-analysis are compromised by the inclusion of the study by Bayindir [15], which was performed solely in patients with osteoarticular brucellosis. It seems premature to extrapolate the results of this study to non-focal brucellosis.

There are very few studies evaluating the efficacy of other regimens and, while the scarce available data do not allow to recommend these regimens, they could improve compliance and facilitate administration of treatment as they involve oral administration and are of similar duration to those already in use. Thus, a combined regimen of co-trimoxazole and doxycycline, for example, yielded good results in the two studies in which it was used in adults [39], [42]. Combined rifampicin-co-trimoxazole yielded good results in children [30], [43], although this regimen poses the risk of inducing resistance to rifampicin and did not yield good results in adults [42].

Since the end of the 1940s, when combined antibiotics began to be used to treat human brucellosis, monotherapy was relegated to a second place and its use has been repeatedly rejected in many publications [14]. Although it is true that co-trimoxazole, rifampicin or quinolones in monotherapy have not yielded good results, this conclusion cannot be extended to doxycycline. The study by Montejo *et al.* [25] reported results with doxycycline in monotherapy similar to those obtained with other regimens recommended by the World Health Organization. Despite this, no other studies have been performed with doxycycline in monotherapy and, consequently, these results cannot be compared with new data.

Although the meta-analysis by Skalsky *et al.* [14] includes seven studies involving monotherapy, they had different characteristics. It compared monotherapy and combined therapy with a similar duration of treatment but combines studies with very different monotherapies such as co-trimoxazole or tetracyclines. This author also rejects monotherapy with tetracyclines based on two studies: the study by Montejo *et al.* [25], mentioned previously and the study by Feiz *et al.* [46], which was not comparable to the former because of the previously explained factors. In spite of this, the relative risks (RR) described are not statistically significant compared with monotherapy using tetracyclines [RR for the combined variable of relapse and treatment failure of 1.01 (CI95% = 0.58–1.77) and RR for therapeutic failure of 0.25 (CI95% = 0.03–2.32)]. Therefore we feel that the use of doxycycline alone in the treatment of human brucellosis cannot be dismissed on the basis of current data, and that there is a need for further studies involving this regimen.

Since only a very small number of studies compared similar regimens administered over different periods, it is difficult to draw any conclusions concerning treatment duration. A duration of 45 days was superior to 30 days for combined doxycycline-gentamicin. For both the doxycycline-streptomycin regimen and the doxycycline-rifampicin regimen, most authors continued treatment for 45 days. The data from these studies suggest that shorter treatment periods yield inferior results, while longer periods do not offer clear advantages (Table 2). Consequently, 6 weeks seems advisable. In a retrospective study [47], an intermittent treatment regimen was reported, but there are no studies to support this regimen.

So far, no studies have examined the cost-efficiency of treatments of human brucellosis. Most studies conducted before the 1990s were conducted in Western countries where preventive measures have succeeded in eradicating the disease and where a health system is in place with sufficient resources for patient care. Currently, brucellosis has become a disease in countries with a lower capacity for medical assistance [1], [63] as a result of the scarcity of healthcare professionals and resources to cover the rural areas and the characteristics of the population often nomadic. These circumstances could account for the greater use of oral drug regimens. Similarly, the difficulty of ensuring satisfactory administration of treatment and adequate follow-up because of economic or cultural reasons could account for the high percentage of studies involving initial treatment in patients following hospitalization, which results in a considerable increase in expenditure.

In 2002, Straight and Martin [67] carried out a review in which they reported the cost of the various drugs used to treat human brucellosis, as well as the associated contraindications and side effects. According to this review, trimethoprim-sulfamethoxazole is the least expensive drug regimen, with a cost of USD 11.02 for 45 days of treatment, followed by doxycycline at USD 12.71 for 45 days of treatment. The most expensive drugs were the quinolones, with an approximate treatment cost of USD 224.06 for 45 days of treatment. Gentamicin was less expensive than streptomycin, although for these two drugs the cost of parenteral administration equipment must be added. The costs of treatment with these drugs in Spain are shown in Table 4. The least expensive combination of drugs is doxycycline and co-trimoxazole which, despite yielding good results in the few clinical trials in which it was used and being considered a cost-efficient combination by some authors [68], has not achieved widespread use. Treatment with doxycycline as monotherapy costs half as much as the least expensive combination, even if treatment is prolonged beyond 45 days. It would be useful to carry out further studies to determine the efficacy of doxycycline monotherapy in various groups of patients.

**Table 4:** Cost of antimicrobial agents for treatment of human brucellosis in Spain [72].

Cost of antimicrobial agents				
Antibiotic	Time of therapy	Dairy dosage (adults)	Dairy cost	Total cost of therapy
Doxycycline	45 days	200 mg	0.48 €	21.6 €
Tetracycline	45 days	2 g	1.16 €	52.2 €
Rifampicin	45 days	600–900 mg	0.78–1.17 €	35.1–52.65 €
Streptomycin*	14–21 days	1 g	2.17 €*	30.38–45.57 €*
Gentamicin*	7 days	5 mg/kg	3.62 €*	25.34 €*
Cotrimoxazole	45 days	480 mg/2400 mg	0.47 €	20.92 €
Ofloxacin	45 days	400 mg	1.36 €	61.22 €

Cost of combined regimens	
Regimen	Cost
Doxycycline 45 days +cotrimoxazole 45 days	42.52 €
Doxycycline 45 days + gentamicin 7 days	46.94 €*
Doxycycline 45 days + streptomycin 14 days	51.98 €*
Doxycycline 45 days + rifampicin 45 days	56.70–74.25 €
Doxycycline 45 days + ofloxacin 45 days	82.82 €
Ofloxacin 45 days + rifampicin 45 days	96.32–113.87 €

* This cost does not include the cost of syringes or intravenous equipment.

It would also prove useful to determine the cost-efficiency of the different alternatives for the treatment of human brucellosis, taking other factors into consideration such as availability of staff and healthcare centers in the various regions and side effects. Studies undertaken to determine the best form of treatment for these patients should take into account social and economic factors [69], and it is difficult to conduct such studies from the perspective of countries where drug administration, follow-up, or availability of antibiotics pose a problem.

The main limitation of any review of treatment for human brucellosis is the scarcity of well-designed clinical trials. Only two of the studies reviewed were in fact double-blind [18], [32] and only five had adequate allocation concealment [25], [27], [28], [32], [42]. Some of these studies included only a small number of patients [36], [41], [45], and most studies concerned the two main regimens: combined doxycycline-rifampicin and combined doxycycline-streptomycin. Studies to evaluate other combinations, such as triple therapy, monotherapy with doxycycline, or other combinations such as doxycycline and quinolones, were insufficient in number to allow any definitive conclusionson their usefulness. In addition, factors such as epidemiological conditions, different criteria and type of clinical monitoring can influence the relapse rate detected. Well design clinical trials are necessary to detect differences in relapses between treatment groups that we have not been able to find in this meta-analysis.

Another limitation is that of the degree in which the recommendations are applicable. The fact that brucellosis is endemic in developing countries means that, in many cases, treatment choice is based on convenience with regard to socio-economic factors, even at the expense of a slight increase in relapse rates [64]. Brucellosis is a disease with low mortality, with a high cure rate for oral treatment, and with episodes of relapse that normally respond well to a further course of the same antibiotics. Consequently, factors such as treatment costs should be taken into consideration in future studies.

In addition, there are no studies to investigate the efficacy of the various regimens depending on patient conditions, complications or relapse risk. The published studies do not consider the possible inclusion of patients with different risk profiles for poor outcome or treatment inefficiency. Ariza *et al.* [70] carried out a study in which the following independent risk factors for relapse were identified: treatment with a “less effective” antibiotic, positive blood culture at the start of treatment, duration of illness of 10 days or less before the start of treatment, male gender and platelet count of 150×10^3^/ml or less. Solera *et al.* published a study [71] in which three risk factors prior to treatment were identified as predictive of a poor clinical outcome: fever of 38.3°C or more, positive blood tests at the outset, and presence of symptoms for less than 10 days at the start of treatment. A model may thus be defined in which patients may be divided into three different groups according to risk of relapse: first, a low-risk group having at least two of the aforementioned factors with a risk of relapse within the year of 4.5%; second, an intermediate-risk group (with two of the factors mentioned) having a risk of relapse within the year of 31.9%; third, a high-risk group, with three of the factors mentioned, having a risk of relapse of 66.7%. There have been no studies with this stratification of patients into risk groups to evaluate the efficacy of treatment for human brucellosis. It is likely that patients with a lower risk of relapse are candidates for simpler and shorter treatment, including monotherapy. This would be an interesting field of research that could change the treatment of brucellosis, and it would be extremely useful primarily in countries with limited resources where the rationalization thereof bears the greatest importance.

Thus, we may conclude that we still face important challenges in the investigation of treatment for human brucellosis. In view of current data, we can say that the most effective regimen is combined doxycycline for 45 days with streptomycin for 14 days or gentamycin 7 days. The alternative is combined doxycycline and rifampicin. Other combinations such as ofloxacin and rifampicin have similar efficacy as well as fewer side effects. However they are more expensive. Combined doxycycline-co-trimoxazole could also offer a low-cost alternative. We do not feel that triple therapy with doxycycline-rifampicin-aminoglycoside can be recommended as the best regimen in view of current data. Since brucellosis affects mainly developing countries and mobilizes economic resources in terms of drugs, other medical supplies and hospital stays, it is even more important to find simple and inexpensive medical solutions. Among these solutions, monotherapy with doxycycline should not be dismissed, especially in patients with a low risk of relapse. For future studies on this issue, we suggest a more personalized treatment according to patient characteristics and risk of relapse.

## Supporting Information

Figure S1 **Funnel plot of comparisons doxycycline-streptomycin vs doxycycline-rifampicin, and quinolone-rifampicin vs doxycycline rifampicin.**
(DOC)

Table S1 **PRISMA checklist.**
(DOC)

Table S2 **list of excluded studies.**
(DOC)

Table S3 **Risk of bias of individual included studies.**
(DOC)

Table S4 **Comparative trials in treatment of human brucellosis since 1985.**
(DOC)

Table S5 **Non-comparative trials in treatment of human brucellosis since 1985.**
(DOC)

Text S1 **Review protocol.**
(DOC)
