# Supplementary material for: Systematic Review and Meta-Analysis of Randomized Clinical Trials in the Treatment of Human Brucellosis
Source: PLoS One. 2012 Feb 29;7(2):e32090. doi: 10.1371/journal.pone.0032090 (PMC3290537; doi:10.1371/journal.pone.0032090)

Figure S1: funnel plot of comparisons doxycycline-streptomycin vs doxycycline-rifampicin, and quinolone-rifampicin vs doxycycline rifampicin

Funnel plot of comparison: Doxycycline and streptomycin vs doxycycline and rifampicin, outcome: Relapses and treatment failure.


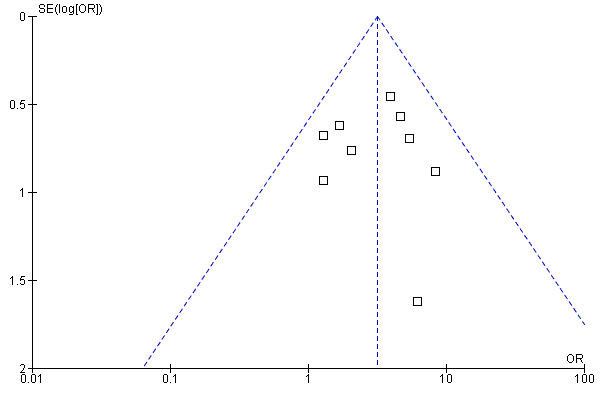


Funnel plot of comparison: Quinolone-rifampicin vs doxycycline-rifampicin, outcome: Relapses and treatment failure.


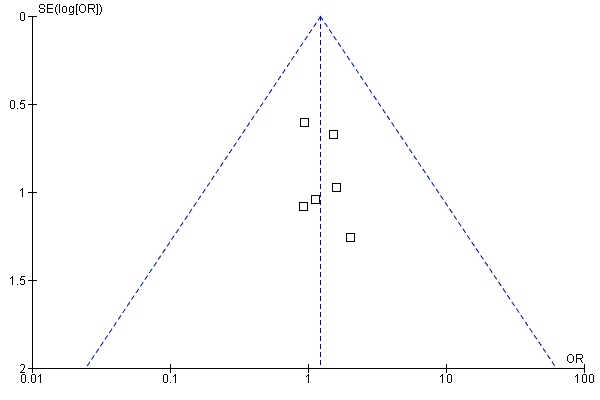

Supplement: Figure S1 — Funnel plot of comparisons doxycycline-streptomycin vs doxycycline-rifampicin, and quinolone-rifampicin vs doxycycline rifampicin. (DOC) [file pone.0032090.s001.doc]
