# Supplementary material for: Systematic Review and Meta-Analysis of Randomized Clinical Trials in the Treatment of Human Brucellosis
Source: PLoS One. 2012 Feb 29;7(2):e32090. doi: 10.1371/journal.pone.0032090 (PMC3290537; doi:10.1371/journal.pone.0032090)
Supplement: Table S2 — list of excluded studies. (DOC) [file pone.0032090.s003.doc]

**Table S2: List of excluded studies**

| **Nº** | **Author and year** | **Reason for exclusion** |
| --- | --- | --- |
| 1 | Suslina ZA et al 2010 | Not a study of brucellosis |
| 2 | Soboleva LA et al 2010 | Not a study of brucellosis |
| 3 | Miki K et al 2007 | Not a study of brucellosis |
| 4 | Lõivukene K et al 2005 | Not a study of brucellosis |
| 5 | Eder L et al 2005 | Not a study of brucellosis |
| 6 | Ozaras R et al 2004 | Not a study of brucellosis |
| 7 | Sekino S et al 2003 | Not a study of brucellosis |
| 8 | Collaert B et al 1993 | Not a study of brucellosis |
| 9 | Grilló MJ et al 2008 | Veterinarian study |
| 10 | Fiorentino MA et al 2008 | Veterinarian study |
| 11 | Diptee MD et al 2007 | Veterinarian study |
| 12 | Stoffregen WC et al 2006 | Veterinarian study |
| 13 | Wanke MM et al 2006 | Veterinarian study |
| 14 | Blasco JM et al 2005 | Veterinarian study. Letter. |
| 15 | Diptee MD et al 2005 | Veterinarian study |
| 16 | Fosgate GT et al 2003 | Veterinarian study |
| 17 | El-Abasy M et al 2003 | Veterinarian study |
| 18 | Olsen SC et al 2003 | Veterinarian study |
| 19 | Kreeger TJ et al 2002 | Veterinarian study |
| 20 | Biancifiori F et al 2000 | Veterinarian study |
| 21 | Geong M et al 2000 | Veterinarian study |
| 22 | Olsen SC et al 2000 | Veterinarian study |
| 23 | Olsen SC et al 2000 | Veterinarian study |
| 24 | Uza FA et al 2000 | Veterinarian study |
| 25 | Samartino LE et al 2000 | Veterinarian study |
| 26 | Lord VR et al 1998 | Veterinarian study |
| 27 | Olsen SC et al 1997 | Veterinarian study |
| 28 | Cheville NF et al 1994 | Veterinarian study |
| 29 | Mustafa AA et al 1993 | Veterinarian study |
| 30 | Smagina AN et al 2010 | Not a study of antibiotic therapy |
| 31 | Apostolou et al 2009 | Not a study of antibiotic therapy |
| 32 | Parker TM et al 2007 | Not a study of antibiotic therapy |
| 33 | Makis AC et al 2005 | Not a study of antibiotic therapy |
| 34 | Aydin M et al 2005 | Not a study of antibiotic therapy. Diagnostic study. |
| 35 | Irmak H et al 2003 | Not a study of antibiotic therapy |
| 36 | Strady A et al 1992 | Not a study of antibiotic therapy |
| 37 | Colmenero JD et al 1992 | Not a study of antibiotic therapy |
| 38 | Gómez Rodriguez N et al | Not a study of antibiotic therapy. Spondylitis. Retrospective. |
| 39 | Nikitiuk NM et al 1987 | Not a study of antibiotic therapy. Diagnostic study. |
| 40 | Mukovozova LA 1986 | Not a study of antibiotic therapy |
| 41 | Mukovozova LA 1986 | Not a study of antibiotic therapy |
| 42 | Boura P et al 1999 | Not a study of antibiotic therapy |
| 43 | Mathai E et al 1996 | Not a study of antibiotic therapy. Diagnostic study. |
| 44 | Lieberman JM et al 2009 | Review |
| 45 | Skalsky K et al 2008 | Review |
| 46 | Al-Tawfiq JA 2008 | Review |
| 47 | Hill Gaston JS et al 2003 | Review |
| 48 | Brumfitt W et al 1994 | Review |
| 49 | Lang R et al 1992 | Review |
| 50 | Burman LG 1986 | Review |
| 51 | Solera J et al 1994 | Review |
| 52 | Sari I et al 2008 | Not a comparative randomized study. Retrospective study |
| 53 | Keles C et al 2001 | Not a comparative randomized study. Case report. Brucellar endocarditis. |
| 54 | Abramson O et al 1997 | Not a randomized study comparing treatment. |
| 55 | Pozdniakova VP et al 1997 | Not a comparative randomized study. Review? |
| 56 | Solera J et al 1997 | Not a randomized study comparing treatment. |
| 57 | Solera J et al 1996 | Not a randomized study comparing treatment. |
| 58 | Khuri-Bulos NA et al 1993 | Not a randomized study comparing treatment. |
| 59 | Cisneros JM et al 1990 | Not a randomized study comparing treatment. |
| 60 | El Idrissi AH et al 2001 | Experimental laboratory study |
| 61 | Galdiero E et al 2000 | Experimental laboratory study |
| 62 | Landínez R et al 1992 | Laboratory study. |
| 63 | Mukovozova LA 1988 | Laboratory study |
| 64 | Mukovozova LA 1987 | Laboratory study |
| 65 | Zheludkov MM et al 1998 | Laboratory study. Diagnostic study. |
| 66 | Liu JB et al 1997 | Traditional Chinese medicine survey. |
| 67 | Sheng YF et al 1993 | Traditional Chinese medicine survey. |
| 68 | Alp E at al 2006 | Osteoarticular brucellosis. Spinal brucellosis. |
| 69 | El Miedany YM et al 2003 | Osteoarticular brucellosis. Spinal brucellosis. |
| 70 | Bayindir Y et al 2003 | Osteoarticular brucellosis. Spinal brucellosis. |
| 71 | Colmenero JD et al 1997 | Osteoarticular brucellosis. Spinal brucellosis. No comparative randomized study. |
| 72 | Faria F et al 1995 | Osteoarticular brucellosis. Spinal brucellosis. Cases report. |
| 73 | Solera J et al 1993 | Osteoarticular brucellosis. |
| 74 | Reguera JM et al 2003 | Brucellar endocarditis |
| 75 | Rodriguez Zapata M et al 1987 | Included in other study |
| 76 | Solera J et al 1992 | Included in other study |
| 77 | Rodriguez M et al 1986 | Included in other study |
| 78 | Rodriguez M et al 1985 | Included in other study |
| 79 | Feng YM 1994 | Not found. |

| **Nº** | **Reference** |
| --- | --- |
| 1 | Suslina ZA, Romantsov MG, Kovalenko AL, Klocheva EG, Rumintseva SA, Bagnenko SF, Piradov MA, Semke VIa, Sukhanov DS. [Therapeutic efficiency of cytoflavine solution for infusion in emergency conditions]. Klin Med (Mosk). 2010;88(4):61-7. |
| 2 | Soboleva LA, Siakin RR, Blinnikova EN, Shul'diakov AA, Pichugina LM. [Parodontitis immunotropic therapy in patients with chronic viral and bacterial infections]. Stomatologiia (Mosk). 2010;89(3):20-2. |
| 3 | Miki K, Urita Y, Ishikawa F, Iino T, Shibahara-Sone H, Akahoshi R, Mizusawa S, Nose A, Nozaki D, Hirano K, Nonaka C, Yokokura T. Effect of Bifidobacterium bifidum fermented milk on Helicobacter pylori and serum pepsinogen levels in humans. J Dairy Sci. 2007 Jun;90(6):2630-40. |
| 4 | Lõivukene K, Pähkla ER, Koppel T, Saag M, Naaber P. The microbiological status of patients with periodontitis in southern Estonia after non-surgical periodontal therapy. Stomatologija. 2005;7(2):45-7. |
| 5 | Eder L, Zisman D, Rozenbaum M, Rosner I. Clinical features and aetiology of septic arthritis in northern Israel. Rheumatology (Oxford). 2005 Dec;44(12):1559-63. |
| 6 | Ozaras R, Tahan V, Mert A, Uraz S, Kanat M, Tabak F, Avsar E, Ozbay G, Celikel CA, Tozun N, Senturk H. The prevalence of hepatic granulomas in chronic hepatitis C. J Clin Gastroenterol. 2004 May-Jun;38(5):449-52. |
| 7 | Sekino S, Ramberg P, Uzel NG, Socransky S, Lindhe J. Effect of various chlorhexidine regimens on salivary bacteria and de novo plaque formation. J Clin Periodontol. 2003 Oct;30(10):919-25. |
| 8 | Collaert B, Edwardsson S, Attström R, Hase JC, Aström M. Microbiology of early supragingival plaque development after delmopinol treatment. Oral Microbiol Immunol. 1993 Feb;8(1):36-41. |
| 9 | Grilló MJ, Marín CM, Barberán M, de Miguel MJ, Laroucau K, Jacques I, Blasco JM. Efficacy of bp26 and bp26/omp31 B. melitensis Rev.1 deletion mutants against Brucella ovis in rams. Vaccine. 2009 Jan 7;27(2):187-91. |
| 10 | Fiorentino MA, Campos E, Cravero S, Arese A, Paolicchi F, Campero C, Rossetti O. Protection levels in vaccinated heifers with experimental vaccines Brucella abortus M1-luc and INTA 2. Vet Microbiol. 2008 Dec 10;132(3-4):302-11. |
| 11 | Diptee MD, Asgarali Z, Campbell M, Fosgate G, Adesiyun AA. Post-exposure serological and bacteriological responses of water buffalo (Bubalus bubalis) to Brucella abortus biovar 1 following vaccination with Brucella abortus strain RB51. Rev Sci Tech. 2007 Dec;26(3):669-78. |
| 12 | Stoffregen WC, Olsen SC, Bricker BJ. Parenteral vaccination of domestic pigs with Brucella abortus strain RB51. Am J Vet Res. 2006 Oct;67(10):1802-8. |
| 13 | Wanke MM, Delpino MV, Baldi PC. Use of enrofloxacin in the treatment of canine brucellosis in a dog kennel (clinical trial). Theriogenology. 2006 Oct;66(6-7):1573-8. |
| 14 | Blasco JM, Moriyon I. Protection of Brucella abortus RB51 revaccinated cows. Comp Immunol Microbiol Infect Dis. 2005 Sep-Nov;28(5-6):371-3 |
| 15 | Diptee MD, Adesiyun AA, Asgarali Z, Campbell M, Adone R. Serologic responses, biosafety and clearance of four dosages of Brucella abortus strain RB51 in 6-10 months old water buffalo (Bubalus bubalis). Vet Immunol Immunopathol. 2006 Jan 15;109(1-2):43-55. |
| 16 | Fosgate GT, Adesiyun AA, Hird DW, Johnson WO, Hietala SK, Schurig GG, Ryan J, Diptee MD. Evaluation of brucellosis RB51 vaccine for domestic water buffalo (Bubalus bubalis) in Trinidad. Prev Vet Med. 2003 May 15;58(3-4):211-25. |
| 17 | El-Abasy M, Motobu M, Sameshima T, Koge K, Onodera T, Hirota Y. Adjuvant effects of sugar cane extracts (SCE) in chickens. J Vet Med Sci. 2003 Jan;65(1):117-9. |
| 18 | Olsen SC, Jensen AE, Stoffregen WC, Palmer MV. Efficacy of calfhood vaccination with Brucella abortus strain RB51 in protecting bison against brucellosis. Res Vet Sci. 2003 Feb;74(1):17-22. |
| 19 | Kreeger TJ, DeLiberto TJ, Olsen SC, Edwards WH, Cook WE. Safety of Brucella abortus strain RB51 vaccine in non-target ungulates and coyotes. J Wildl Dis. 2002 Jul;38(3):552-7. |
| 20 | Biancifiori F, Garrido F, Nielsen K, Moscati L, Durán M, Gall D. Assessment of a monoclonal antibody-based competitive enzyme linked immunosorbent assay (cELISA) for diagnosis of brucellosis in infected and Rev. 1 vaccinated sheep and goats. New Microbiol. 2000 Oct;23(4):399-406. |
| 21 | Geong M, Robertson ID. Response of Bali cattle (Bos javanicus) to vaccination with Brucella abortus strain 19 in West Timor. Prev Vet Med. 2000 Nov 16;47(3):177-86. |
| 22 | Olsen SC. Responses of adult cattle to vaccination with a reduced dose of Brucella abortus strain RB51. Res Vet Sci. 2000 Oct;69(2):135-40. |
| 23 | Olsen SC. Immune responses and efficacy after administration of a commercial Brucella abortus strain RB51 vaccine to cattle. Vet Ther. 2000 Summer;1(3):183-91. |
| 24 | Uza FA, Samartino L, Schurig G, Carrasco A, Nielsen K, Cabrera RF, Taddeo HR. Effect of vaccination with Brucella abortus strain RB51 on heifers and pregnant cattle. Vet Res Commun. 2000 Apr;24(3):143-51. |
| 25 | Samartino LE, Fort M, Gregoret R, Schurig GG. Use of Brucella abortus vaccine strain RB51 in pregnant cows after calfhood vaccination with strain 19 in Argentina. Prev Vet Med. 2000 Jun 12;45(3-4):193-9. |
| 26 | Lord VR, Cherwonogrodzky JW, Schurig GG, Lord RD, Marcano MJ, Meléndez GE. Venezuelan field trials of vaccines against brucellosis in swine. Am J Vet Res. 1998 May;59(5):546-51. |
| 27 | Olsen SC, Cheville NF, Kunkle RA, Palmer MV, Jensen AE. Bacterial survival, lymph node pathology, and serological responses of bison (Bison bison) vaccinated with Brucella abortus strain RB51 or strain 19. J Wildl Dis. 1997 Jan;33(1):146-51. |
| 28 | Cheville NF, Jensen AE, Morfitt DC, Stabel TJ. Cutaneous delayed hypersensitivity reactions of cattle vaccinated with mutant strains of Brucella abortus, using brucellins prepared from various brucellar strains. Am J Vet Res. 1994 Sep;55(9):1261-6. |
| 29 | Mustafa AA, Abusowa M. Field-oriented trial of the Chinese Brucella suis strain 2 vaccine on sheep and goats in Libya. Vet Res. 1993;24(5):422-9. |
| 30 | Smagina AN, Lin'kova IuN, Shul'diakov AA, Liapina EP. [Cycloferon in complex therapy of chronic brucellosis]. Antibiot Khimioter. 2010;55(7-8):42-5. |
| 31 | Apostolou F, Gazi IF, Kostoula A, Tellis CC, Tselepis AD, Elisaf M, Liberopoulos EN. Persistence of an atherogenic lipid profile after treatment of acute infection with Brucella. J Lipid Res. 2009 Dec;50(12):2532-9. |
| 32 | Parker TM, Murray CK, Richards AL, Samir A, Ismail T, Fadeel MA, Jiang J, Wasfy MO, Pimentel G. Concurrent infections in acute febrile illness patients in Egypt. Am J Trop Med Hyg. 2007 Aug;77(2):390-2. |
| 33 | Makis AC, Galanakis E, Hatzimichael EC, Papadopoulou ZL, Siamopoulou A, Bourantas KL. Serum levels of soluble interleukin-2 receptor alpha (sIL-2Ralpha) as a predictor of outcome in brucellosis. J Infect. 2005 Oct;51(3):206-10. |
| 34 | Aydin M, Fuat Yapar A, Savas L, Reyhan M, Pourbagher A, Turunc TY, Ziya Demiroglu Y, Yologlu NA, Aktas A. Scintigraphic findings in osteoarticular brucellosis. Nucl Med Commun. 2005 Jul;26(7):639-47. |
| 35 | Irmak H, Buzgan T, Karahocagil MK, Evirgen O, Akdeniz H, Demiröz AP. The effect of levamisole combined with the classical treatment in chronic brucellosis. Tohoku J Exp Med. 2003 Dec;201(4):221-8. |
| 36 | Strady A, Lienard M, Gillant JC, Barrat F, Poncelet S, Laudat P, Audurier A, Limet J, Ajjan N. [Brucella vaccination in professionally exposed subjects. Prospective study]. Presse Med. 1992 Sep 19;21(30):1408-12. |
| 37 | Colmenero JD, Cisneros JM, Orjuela DL, Pachón J, Garcia-Portales R, Rodriguez-Sampedro F, Juarez C. Clinical course and prognosis of Brucella spondylitis. Infection. 1992 Jan-Feb;20(1):38-42. |
| 38 | Gómez Rodríguez N, Martínez Vázquez C, Sopeña B, Portela J, Carbajo M, Guerra V, Arasa X, Barrio Gómez E. [Non-tuberculous spondylodiscitis. Multicenter study of 19 cases]. Rev Clin Esp. 1989 Apr;184(6):289-96. |
| 39 | Nikitiuk NM, Nefedova LA, Pavlova LI, Amiriev SA, Kurmanova KB. [Comparative study of the diagnostic value of Brucella allergens in a controlled epidemiological trial]. Zh Mikrobiol Epidemiol Immunobiol. 1987 Feb;(2):76-80. |
| 40 | Mukovozova LA. [Effectiveness of levamisole in the complex treatment of patients with active forms of brucellosis]. Klin Med (Mosk). 1986 Mar;64(3):47-51. |
| 41 | Mukovozova LA. [Comparative effectiveness of levamisole and vaccine in the complex treatment of patients with brucellosis]. Ter Arkh. 1986;58(10):43-5. |
| 42 | Boura P, Skendros P, Kountouras J, Zacharioudaki E, Tsapas G. Effect of bacterial extracts on the inmunologic profile in chronic relapsing brucellosis patients. Int J Inmunopathol Pharmacol. 1999; 12:103-111 |
| 43 | Mathai E, Singhal A, Verghese S, D'Lima D, Mathai D, Ganesh A, et al. Evaluation of an ELISA for the diagnosis of brucellosis. Indian J Med Research. 1996; 103: 323-4. |
| 44 | Lieberman JM. North American zoonoses. Pediatr Ann. 2009 Apr;38(4):193-8. |
| 45 | Skalsky K, Yahav D, Bishara J, Pitlik S, Leibovici L, Paul M. Treatment of human brucellosis: systematic review and meta-analysis of randomised controlled trials. BMJ. 2008 Mar 29;336(7646):701-4. |
| 46 | Al-Tawfiq JA. Therapeutic options for human brucellosis. Expert Rev Anti Infect Ther. 2008 Feb;6(1):109-20. |
| 47 | Hill Gaston JS, Lillicrap MS. Arthritis associated with enteric infection. Best Pract Res Clin Rheumatol. 2003 Apr;17(2):219-39. |
| 48 | Brumfitt W, Hamilton-Miller JM. Limitations of and indications for the use of co-trimoxazole. J Chemother. 1994 Feb;6(1):3-11. |
| 49 | Lang R, Rubinstein E. Quinolones for the treatment of brucellosis. J Antimicrob Chemother. 1992 Apr;29(4):357-60. |
| 50 | Burman LG. Significance of the sulfonamide component for the clinical efficacy of trimethoprim-sulfonamide combinations. Scand J Infect Dis. 1986;18(2):89-99. |
| 51 | Solera J, Martínez-Alfaro E, Sáez L. [Meta-analysis of the eficacy on the combination of rifampicin and doxycycline in the treatment of human brucellosis]. Med Clin (Barc). 1994; 102: 731-8. |
| 52 | Sari I, Altuntas F, Hacioglu S, Kocyigit I, Sevinc A, Sacar S, Deniz K, Alp E, Eser B, Yildiz O, Kaynar L, Unal A, Cetin M. A multicenter retrospective study defining the clinical and hematological manifestations of brucellosis and pancytopenia in a large series: Hematological malignancies, the unusual cause of pancytopenia in patients with brucellosis. Am J Hematol. 2008 Apr;83(4):334-9. |
| 53 | Keleş C, Bozbuğa N, Sişmanoğlu M, Güler M, Erdoğan HB, Akinci E, Yakut C. Surgical treatment of Brucella endocarditis. Ann Thorac Surg. 2001 Apr;71(4):1160-3. |
| 54 | Abramson O, Abu-Rashid M, Gorodischer R, Yagupsky P. Failure of short antimicrobial treatments for human brucellosis. Antimicrob Agents Chemother. 1997 Jul;41(7):1621-2. |
| 55 | Pozdniakova VP, Nestetrova LIa, Smirnova LB, Iakovlev VP, Blatun LA, Koshil' OI, Fedorov SM, Bakalova LA, Strachunskiĭ LS, Kurmanova KB. [Pefloxacin mesylate--clinical effectiveness in various forms of infectious-inflammatory diseases]. Antibiot Khimioter. 1997;42(7):20-3. |
| 56 | Solera J, Espinosa A, Martínez-Alfaro E, Sánchez L, Geijo P, Navarro E, Escribano J, Fernández JA. Treatment of human brucellosis with doxycycline and gentamicin. Antimicrob Agents Chemother. 1997 Jan;41(1):80-4. |
| 57 | Solera J, Espinosa A, Geijo P, Martínez-Alfaro E, Sáez L, Sepúlveda MA, Ruiz-Ribó MD. Treatment of human brucellosis with netilmicin and doxycycline. Clin Infect Dis. 1996 Mar;22(3):441-5. |
| 58 | Khuri-Bulos NA, Daoud AH, Azab SM. Treatment of childhood brucellosis: results of a prospective trial on 113 children. Pediatr Infect Dis J. 1993 May;12(5):377-81. |
| 59 | Cisneros JM, Viciana P, Colmenero J, Pachón J, Martinez C, Alarcón A. Multicenter prospective study of treatment of Brucella melitensis brucellosis with doxycycline for 6 weeks plus streptomycin for 2 weeks. Antimicrob Agents Chemother. 1990 May;34(5):881-3. |
| 60 | el Idrissi AH, Benkirane A, el Maadoudi M, Bouslikhane M, Berrada J, Zerouali A. Comparison of the efficacy of Brucella abortus strain RB51 and Brucella melitensis Rev. 1 live vaccines against experimental infection with Brucella melitensis in pregnant ewes. Rev Sci Tech. 2001 Dec;20(3):741-7. |
| 61 | Galdiero E, Romano Carratelli C, Vitiello M, Nuzzo I, Del Vecchio E, Bentivoglio C, Perillo G, Galdiero F. HSP and apoptosis in leukocytes from infected or vaccinated animals by Brucella abortus. New Microbiol. 2000 Jul;23(3):271. |
| 62 | Landínez R, Liñares J, Loza E, Martínez-Beltrán J, Martín R, Baquero F. In vitro activity of azithromycin and tetracycline against 358 clinical isolates of Brucella melitensis. Eur J Clin Microbiol Infect Dis. 1992 Mar;11(3):265-7. |
| 63 | Mukovozova LA. [Effect of levamisole on the cellular immunity indices of patients with active forms of brucellosis]. Zh Mikrobiol Epidemiol Immunobiol.1988 Jun;(6):61-5. |
| 64 | Mukovozova LA. [Dynamics of the humoral immunity indices in patients with subacute brucellosis being treated with levamisole]. Zh Mikrobiol Epidemiol Immunobiol. 1987 Jul;(7):56-9. |
| 65 | Zheludkov MM, Kulakov IuK. [Use of brucellar protein antigen synthesitzed in Escherichia coli cells in the enzyme immunoassay]. Zhurnal mikrobiologii, epidemiologii, i immunobiologii. 1998; 5: 74-7. |
| 66 | Liu JB, Zhou WC, Wang QZ. [Clinical and experimental studies of supplemented sini san in treating chronic brucellosis]. Zhongguo Zhong Xi Yi Jie He Za Zhi. 1997 Aug;17(8):470-2. |
| 67 | Sheng YF, Zhao GC, Hu SH. [Observations on treatment of chronic brucellosis with combined TCM-WM therapy]. Chinese Journal of integrated Traditional and Western Medicine. 1993; 13: 88-90. |
| 68 | Alp E, Koc RK, Durak AC, Yildiz O, Aygen B, Sumerkan B, Doganay M. Doxycycline plus streptomycin versus ciprofloxacin plus rifampicin in spinal brucellosis [ISRCTN31053647]. BMC Infect Dis. 2006 Apr 11;6:72. |
| 69 | El Miedany YM, El Gaafary M, Baddour M, Ahmed I. Human brucellosis: do we need to revise our therapeutic policy? J Rheumatol. 2003 Dec;30(12):2666-72. |
| 70 | Bayindir Y, Sonmez E, Aladag A, Buyukberber N. Comparison of five antimicrobial regimens for the treatment of brucellar spondylitis: a prospective, randomized study. J Chemother. 2003 Oct;15(5):466-71. |
| 71 | Colmenero JD, Jiménez-Mejías ME, Sánchez-Lora FJ, Reguera JM, Palomino-Nicás J, Martos F, García de las Heras J, Pachón J. Pyogenic, tuberculous, and brucellar vertebral osteomyelitis: a descriptive and comparative study of 219 cases. Ann Rheum Dis. 1997 Dec;56(12):709-15. |
| 72 | Faria F, Viegas F. Spinal brucellosis: a personal experience of nine patients and a review of the literature. Paraplegia. 1995 May;33(5):294-5. |
| 73 | Solera J, Paulino J, Rodríguez M, Geijo P, Largo J, Grupo GECMEI. [Brucellosis with osteoarticular involvement: must it be treated in a different way?]. Rev Esp Reumatol. 1993; 20 (Supl 1): 153. |
| 74 | Reguera JM, Alarcón A, Miralles F, Pachón J, Juárez C, Colmenero JD. Brucella endocarditis: clinical, diagnostic, and therapeutic approach. Eur J Clin Microbiol Infect Dis. 2003 Nov;22(11):647-50. |
| 75 | Rodriguez Zapata M, Gamo Herranz A, De La Morena Fernández J. Comparative study of two regimens in the treatment of brucellosis. Chemioterapia. 1987 Jun;6(2 Suppl):360-2. |
| 76 | Solera J, Paulino J, Rodríguez Zapata M, Geijó P, Medrano F, Jiménez Zorzo F, et al. [Multicentre comparative therapeutic trial of rifampicin and doxycycline versus streptomycin and doxycycline in human brucellosis. Primary evaluation results]. Rev Esp Reumatol. 1992; 19: 219. |
| 77 | Rodríguez M, Gamo A, de la Morena J. [Evaluation of several regimens for the treatment of brucellosis: rifampicin-doxycycline vs streptomycin-doxycycline. SEMER. 1986; 80: 32-3. |
| 78 | Rodriguez M, Gamo A, de la Morena J. [Assessment of two regimens for the treatment of brucellosis: rifampicin-doxycycline vs streptomycin-doxycycline. Preliminary results].Archivos de la Facultad de Medicina de Zaragoza. 1985; 43: 171 |
| 79 | Feng YM. Randomized controlled trial of six antibacterial methods in brucellosis. Endemic diseases collections. 1994; 15: 66. |

Studies published before 1985

| **Nº** | **Reference** |
| --- | --- |
| 80 | Sumarokov AA, Karinskaia GA, Dranovskaia EA, Vershilova PA, Sharipov MK. [Comparative study of the safety, reactogenicity and antigenic activity of chemical and live brucellosis vaccines in a controlled epidemiological trial]. Zh Mikrobiol Epidemiol Immunobiol. 1984 Feb;(2):58-63. |
| 81 | Vargas V, Pedreira JD, Clotet B, Juste C, Guardia J, Bacardi R. [Treatment of acute brucellosis with cotrimoxazole, doxicyclin and streptomycin. A comparative study (author's transl)]. Med Clin (Barc). 1980 Dec 15;75(10):418-20. |
| 82 | Gross RL. Clinical experiences with levamisole. Adv Exp Med Biol. 1976;73 PT-A:424-7. |
| 83 | D'Alessandro L, Faenza L, Di Palma D, Russo P. [Trimethoprim-sulfamethoxazole (TM-SMZ) combination in the treatment of brucellosis]. Minerva Med. 1974 May 30;65(41):2350-6. |
| 84 | Feiz JM, Sabbaghian H, Sohrabi F. A comparative study of therapeutic agents used for treatment of acute brucellosis. Br J Clin Pract. 1973 Nov;27(11):410-3. |
| 85 | Weeke E, Bendixen G. Evaluation of the primary immune response in uremia and after extracorporeal irradiation of the blood. Acta Med Scand. 1972 Oct;192(4):267-9. |
| 86 | Fiaccadori F, Camilloni R, Ghinelli F, Pizzigoni G. [Comparative study of the use of tetracycline in low dosage in the treatment of brucellosis]. G Clin Med. 1972 Jun;53(6):290-9. |
| 87 | De Rosa F, Fabiani F. [Therapy of human brucellosis with cephalexin]. Clin Ter. 1970 Apr 30;53(2):141-4. |
| 88 | Pacheco G, da Silva DS, da Silva JG. [Use of the new brucellosis curative vaccine]. Hospital (Rio J). 1970 Apr;77(4):1397-405. |
| 89 | McDevitt DG. Ampicillin in the treatment of brucellosis. A controlled therapeutic trial. Br J Ind Med. 1970 Jan;27(1):67-71. |
| 90 | Pappagianis D, Elberg SS, Crouch D. Immunization against Brucella infections. Effects of graded doses of viable attenuated Brucella melitensis in humans. Am J Epidemiol. 1966 Jul;84(1):21-31. |
| 91 | Buzon L, Bouza E, Rodriguez M. Treatment of brucellosis with rifampicin+tetracycline vs TMP/SMZ. A prospective and randomized study. Chemioterapia. 1982; 1 (4 Suppl): 221. |
| 92 | Murray MJ, Murray AB, Murray MB, Murray CJ. The adverse effect of iron repletion on the course of certain infections. BMJ. 1978; 2: 1113-5. |
| 93 | Gong NC, Thavaraja SK, Chan KE, Chai KH. Treatment of urinary infection with cotrimoxazole (trimethoprim sulphamethoxazole) MedJMalaysia. 1974; 28: 194-6. |
