# Supplementary material for: Systematic Review and Meta-Analysis of Randomized Clinical Trials in the Treatment of Human Brucellosis
Source: PLoS One. 2012 Feb 29;7(2):e32090. doi: 10.1371/journal.pone.0032090 (PMC3290537; doi:10.1371/journal.pone.0032090)
Supplement: Table S3 — Risk of bias of individual included studies. (DOC) [file pone.0032090.s004.doc]

**Table S3:** Risk of bias of the different trials included in the systematic review

| **Author and year** | **Random sequence generation (selection bias)** | **Allocation concealment (selection bias)** | **Blinding of participants and personnel** | **Incomplete outcome data (attrition bias)** |
| --- | --- | --- | --- | --- |
| Ariza J 1985 | Unclear | High risk | No | Low risk |
| Ariza J 1985 | Unclear | High risk | No | Low risk |
| Acocella 1989 | Low risk | Unclear | No | Low risk |
| Colmenero JD 1989 | Low risk | High risk | No | Low risk |
| Lubani MM 1989 | Unclear | High risk | No | Low risk |
| Lang R 1990 | Low risk | High risk | No | Low risk |
| Solera J 1991 | Unclear | High risk | No | Low risk |
| Ariza J 1992 | Low risk | Unclear | Yes | Low risk |
| Lang R 1992 | Unclear | Unclear | No | Low risk |
| Montejo JM 1993 | Low risk | Low risk | No | Low risk |
| Akova 1993 | Unclear | High risk | No | Low risk |
| Colmenero JD 1994 | Unclear | High risk | No | Low risk |
| Solera J 1995 | Unclear | High risk | No | Low risk |
| Kalo T 1996 | High risk | High risk | No | Low risk |
| Agalar C 1999 | Unclear | Unclear | No | Low risk |
| Saltoglu N 2002 | High risk | High risk | No | Low risk |
| Solera J 2004 | Low risk | Low risk | Yes | Low risk |
| Karabay O 2004 | Unclear | Unclear | No | Low risk |
| Hasanjani MR 2004 | Low risk | Low risk | No | Low risk |
| Ersoy Y 2005 | Unclear | Unclear | No | Low risk |
| Hasanjani MR 2006 | Low risk | Low risk | No | Low risk |
| Hasanjani MR 2006 | High risk | High risk | No | Low risk |
| Ranjbar M 2007 | Low risk | High risk | No | Unclear |
| Alavi SM 2007 | Unclear | High risk | No | Low risk |
| Keramat F 2009 | Low risk | Unclear | No | Unclear |
| Hasanjani 2010 | Low risk | Low risk | No | Low risk |
