# Supplementary material for: Systematic Review and Meta-Analysis of Randomized Clinical Trials in the Treatment of Human Brucellosis
Source: PLoS One. 2012 Feb 29;7(2):e32090. doi: 10.1371/journal.pone.0032090 (PMC3290537; doi:10.1371/journal.pone.0032090)
Supplement: Table S4 — Comparative trials in treatment of human brucellosis since 1985. (DOC) [file pone.0032090.s005.doc]

**Table S4**: Comparative trials in treatment of human brucellosis since 1985

| **Year[ref]** | **Type of study** | **Follow-up*** | **Hospital admission** | **Regimen of therapy**  **and duration (days)** | **Nº of patients** | **Relapses** | **Failure** | **Relapses+**  **Failure** | **Time to deferv** |
| --- | --- | --- | --- | --- | --- | --- | --- | --- | --- |
| 1985 [8] | RCT | 6-24 | No | TETR ó DX (30)+ STP (21) | 28 | 2 (7.1%) | 0 | 2 (7.1%) | 11.5 |
|  |  |  |  | DX (30)+ RF (30) | 18 | 7 (38.8%) | 0 | 7 (38.8%) | 2.6 |
| 1985 [9] | RCT | 16.4 | No | TETR (21)+STP (14) | 27 | 4 (14.8%) | NR | 4 (14.8%) | 4.2 |
|  |  |  |  | TMP/SMX (45) | 28 | 13(46.6%) | NR | 13 (46.6%) | 8.4 |
| 1989[10] | RCT | 12 | Yes(86ptes) | TETR (21) +STP (14) | 27 | 6 (22.2%) | 5 (18.52%) | 11 (40.72%) | NR |
|  |  |  |  | DX (45) + STP (14) | 53 | 0 | 2 (3.77%) | 2 (3.77%) | NR |
|  |  |  |  | DX (45) + RF (45) | 63 | 3 (4.76%) | 0 | 3 (4.76%) | NR |
| 1989[11] | RCT | 6 | No | DX (30) +STP (21) | 59 | 3 (5%) | 2 (3.4%) | 5 (8.4%) | 3.5 |
|  |  |  |  | DX(45) +RF (45) | 52 | 7 (13.46%) | 0 | 7 (13.46%) | 3.5 |
| 1989 [30] | RCT | 21 | Yes(10 days) | Multiple regimens | 1100 |  |  |  |  |
| 1990 [41] | RCT | 12 | No | CPX (42) | 6 | 5 (83.3%) | 0 | 5 (83.3%) | NR |
|  |  |  |  | DX (42) + RF (42) | 4 | 0 | 0 | 0 | NR |
| 1991 [17] | RCT | 12 | No | DX (45)+STP (14) | 38 | 2 (5.26%) | 1 (2.63%) | 3 (7.89%) | 4.2 |
|  |  |  |  | DX (45) + RF (45) | 38 | 9 (23.68%) | 3 (7.89%) | 12 (31.58%) | 4.4 |
| 1992 [18] | DBRCT | 15.7 | Yes (15 days) | DX (45) +STP (15) | 51 | 2 (3.9%) | 1 (1.96%) | 3 (5.9%) | 3.2 |
|  |  |  |  | DX (45)+ RF (45) | 44 | 3 (6.81%) | 2 (4.5%) | 5 (11.36%) | 4.2 |
| 1992 [45] | RCT | 6 | No | Ceftriaxone (≥14) | 8 | 1 (12.5%) | 6 (75%) | 7 (87.5%) | NR |
|  |  |  |  | DX (28) + STP (14) | 10 | 0 | 0 | 0 | NR |
| 1993 [25] | RCT | 12 | No | DX (42)+STP (14) | 40 | 3 (7.5%) | 0 | 3(7.5%) | NR |
|  |  |  |  | DX(42) + STP(21) | 44 | 1 (2.27%) | 0 | 1 (2.27%) | NR |
|  |  |  |  | DX (28)+ RF(28) | 65 | 14 (21.5%) | 1 (1.5%) | 15 (23%) | NR |
|  |  |  |  | DX (42)+ RF (42) | 46 | 5 (10.86%) | 1 (2.17%) | 6 (13%) | NR |
|  |  |  |  | DX (42) | 71 | 10 (14.08%) | 0 | 10 (14.08%) | NR |
|  |  |  |  | TMP/SMX (180) | 64 | 2 (3.125%) | 4 (6.25%) | 6 (9.37%) | NR |
| 1993 [19] | RCT | 14.6 | Yes (14 days) | DX (42)+RF (42) | 30 | 1 (3.3%) | 0 | 1 (3.3%) | 5.1 |
|  |  |  |  | OFX(42) + RF (42) | 31 | 1 (3.2%) | 1 (3.2%) | 2 (6.4%) | 6.3 |
| 1994 [29] | RCT | 6 | Yes | DX (42)+ STP (21) | 10 | 0 | 0 | 0 | NR |
|  |  |  |  | DX (42) + RF (42) | 10 | 1 (10%) | 1 (10%) | 2 (20%) | NR |
| 1995 [20] | RCT | 12 | No | DX (45)+STP (14) | 94 | 5 (5.3%) | 2 (2.13%) | 7(7.43%) | 4.3 |
|  |  |  |  | DX (45)+RF (45) | 100 | 16 (16%) | 8 (8%) | 24 (24%) | 4.63 |
| 1996 [36] | RCT | 6 | No | DX (42)+RF (42) | 12 | 1 (8.33%) | 0 | 1(8.33%) | 5 |
|  |  |  |  | DX (42)+CPX (42) | 12 | 1 (8.33%) | 0 | 1(8.33%) | 4 |
| 1997 [31] | NRCT | 12 | No | DX (30)+ G (7) | 35 | 8(22.9%) | 0 | 8 (22.9%) | NR |
|  |  |  |  | DX (45) + G (7) | 17 | 1 (5.9%) | 0 | 1(5.9%) | NR |
| 1999 [21] | RCT | 12 | Yes | DX (45)+RF (45) | 20 | 2 (10%) | 0 | 2 (10%) | 3.85 |
|  |  |  |  | CPX (30)+RF(30) | 20 | 3 (15%) | 0 | 3 (15%) | 2.78 |
| 2002 [37] | RCT | 6 | No | DX (45)+RF(45) | 30 | 2 (6.7%) | NR | 2 (6.7%) | NR |
|  |  |  |  | OFX (45) + RF(45) | 27 | 2 (7.3%) | NR | 2 (7.3%) | NR |
| 2004 [32] | DBRCT | 8.7 | No | DX (30)+G (7) | 73 | 15 (20.55%) | NR | 15 (20.55%) | NR |
|  |  |  |  | DX (45) +G (7) | 73 | 9 (12.3%) | NR | 9 (12.3%) | NR |
| 2004 [22] | RCT | ≈5 | Yes (at least 10 days) | DX (45)+RF (45) | 14 | 2 (14.29%) | 0 | 2(14.29%) | 4.4 |
|  |  |  |  | OFX (30) + RF (30) | 15 | 2(13.3%) | 0 | 2 (13.3%) | 3.1 |
| 2004 [42] | RCT | 12 | No | TMP/SMX (60)+RF (60) | 140 | 14 (10%) | 23 (16.4%) | 37 (26.4%) | NR |
|  |  |  |  | DX (60) +TMP/SMX (60) | 140 | 12 (8.6%) | 10 (7.1%) | 22 (15.7%) | NR |
| 2005 [26] | RCT | 6-18 | Yes (some patients)^+^ | DX (42)+STP (21) | 32 | 3 (9.7%) | 1 (3.1%) | 4 (12.5%) | NR |
|  |  |  |  | DX (42) + RF(42) | 45 | 6 (13.3%) | 1 (2.2%) | 7 (15.6%) | NR |
|  |  |  |  | OFX (42) + RF(42) | 41 | 5 (12.8%) | 1(2.4%) | 6(14.6%) | NR |
| 2006 [27] | RCT | 12 | No | DX (45)+STP (14) | 94 | 3 (3.2%) | 4 (4.3%) | 7(7.5%) | NR |
|  |  |  |  | DX (45)+G (7) | 97 | 3 (3.1%) | 2 (2.1%) | 5(5.2%) | NR |
| 2006 [43] | RCT | 12 | No | TMP/SMX (42)+RF (42) | 64 | NR | NR | 7(10.9%) | NR |
|  |  |  |  | TMP/SMX (56) + RF(56) | 66 | NR | NR | 3(4.5%) | NR |
| 2007 [16] | RCT | 6 | Yes(some patients)^+^ | DX (56-84)+RF(56-84)+AMK (7) | 110 | 6 (5.7%) | 4 (3.6%) | 10 (9.1%) | NR |
|  |  |  |  | DX (56)+RF (56) | 110 | 9 (9.3%) | 13 (11.8%) | 22 (20%) | NR |
| 2007 [39] | RCT | 6 | No | DX(56)+RF(56) | 51 | 6 (11.76%) | 5 (9.81%) | 11 (21.56%) | NR |
|  |  |  |  | DX (56) +TMP/SMX (56) | 51 | 3 (5.88%) | 1 (1.94%) | 4 (7.84%) | NR |
| 2009 [38] | RCT | 6 | Yes (some patients)^+^ | DX (56-84)+RF (56-84) | 61 | 2 (7.7%)* | 2 (3.3%) | 4 (6.6%) | <14 |
|  |  |  |  | DX (56-84)+CPX (56-84) | 55 | 7 (17.5%)* | 7(12.7%) | 14 (25.5%) | <14 |
|  |  |  |  | CPX (56-84)+RF (56-84) | 62 | 3 (8.3%) | 3 (4.8%) | 6 (9.7%) | NR |
| 2010 [28] | RCT | 12 | Yes (some patients)^+^ | DX (45)+STP (14) | 82 | 5 (6.1%) | 4 (4.88%) | 9 (10.98%) | NR |
|  |  |  |  | DX(56)+G (5) | 82 | 2 (2.4%) | 2 (2.4%) | 4 (4.8%) | NR |

*The actual follow up time is indicated when reported. If the actual time is not reported we indicate the one that was predicted in the study.

+Outpatients and inpatients were included but the number or percentage of inpatients or the time of admission was not specified.

Abbreviations: RCT= randomized clinical trial; NRCT= nonrandomized clinical trial; DBRCT=double-blind randomized clinical trial. NR=not reported

DX= doxycycline; RF= rifampin; TETR= tetracycline or oxitetracycline; STP=streptomycin; G= gentamicin; AMK= amikacin; TMP/SMX=cotrimoxazole; OFX=ofloxacin; CPX=ciprofloxacin.

Dosage of the used drug was: Doxycycline 200mg daily; Streptomycin 1g daily; Gentamicin 240 mg daily or 5 mg/Kg/day; Tetracycline 2g daily (0.5mg four times a day); Rifampicin 600-1200mg daily or 10-20 mg/Kg/day; TMP/SMX between 160/800 mg /8 hours and 80/400 mg / 12 hours, or trimetroprim 8-10 mg/Kg/day and sulfametoxazole 40-50mg/Kg/day; Ciprofloxacin: 500-1000 mg/12 hours; Ofloxacin: 400mg daily; Ceftriaxone ≤ 2g/day; Amikacin 7.5 mg/12 hours im.
