# Supplementary material for: Systematic Review and Meta-Analysis of Randomized Clinical Trials in the Treatment of Human Brucellosis
Source: PLoS One. 2012 Feb 29;7(2):e32090. doi: 10.1371/journal.pone.0032090 (PMC3290537; doi:10.1371/journal.pone.0032090)
Supplement: Table S5 — Non-comparative trials in treatment of human brucellosis since 1985. (DOC) [file pone.0032090.s006.doc]

**Table S5:** Non-comparative trials in treatment of human brucellosis

| **Year [ref]** | **Type of**  **study** | **Follow-up**  **(months)** | **Hospital admission** | **Regimen of therapy and duration (days)** | **Nº patients** | **Relapse** | **Failure** | **Relapse + failure** | **Time of defervescence** |
| --- | --- | --- | --- | --- | --- | --- | --- | --- | --- |
| 1987 [47] | R | NR | Yes (not clear %) | Minocycline (45-50) | 295 | 10 (3.39%) | NR | 10 (3.39%) | <5 (in 82% of patients) |
| 1989 [52] | NCCT | NR* | NR | Ceftriaxone (7) | 14 | 1 (7.14%) | 5 (35.7%) | 6 (42.9%) | NR |
| 1990 [51] | PNR | 6 | NR* | RF (21)+TMP/SMX (42) | 16 | 0 | 0 | 0 | NR |
|  |  |  |  | TETR (42)+RF (42) | 24 | 0 | 0 | 0 | NR |
|  |  |  |  | TETR (42)+STP (14) | 56 | 1 (1.8%) | 0 | 1 (1.8%) | NR |
|  |  |  |  | RF (21-42) | 10 | 2 (20%) | 0 | 2 (20%) | NR |
| 1990 [24] | NCCT | 6 | No | DX (42)+STP (14) | 139 | 4/102 (3.9%) | 5 (3.6%) | 9 (6.48%) | <7 |
| 1991 [48] | NCCT | 8-24 | No | OFX(21) | 5 | 3 (60%) | 0 | 3 (60%) | 3-5 |
| 1992 [49] | NCCT | 2-11 | No | CPX(21-42) | 14 | 3 (21.4%) | 0 | 3(21.4%) | 6.5 |
| 1992 [50] | NCCT | 24 | No | CPX (42-84) | 16 | 4 (25%) | 1 (6.25%) | 5 (31.25%) | <7 |
| 1996 [33] | NCCT | 9 (1-18) | Yes (some patients) | DX+netilmicina (45) | 64 | 8 (12.5%) | 5 (7.8%) | 13 (21.9%) | 3.9 |
| 1997 [53] | NCCT | 6 | No | DX ó TMP/SMX+G (21) | 15 | 4 (26.7%) | 1 (6.7%) | 5 (33.3%) | 3-5 |
| 2001[34] | NCCT | 12 | No | Azithromycin (21) + G (7) | 10 | 3 (30%) | 2 (20%) | 5 (50%) | NR |
| 2003 [40] | R | 9 | Yes | Minocycline +RF (21) | 239 (174) | 3 (1.7%) | 0 | 3 (1.7%) | 3.5 |

Abbreviations R= retrospective; PNR= prospective nonrandomized; NCCT= non-comparative clinical trial. NR=not reported. DX= doxycycline; RF= rifampicin; TETR= tetracycline or oxitetracycline; STP=streptomycin; TMP/SMX=cotrimoxazole; OFX=ofloxacin; CPX=ciprofloxacin.

Dosage of the used drugs was: Doxycycline 200mg daily; Streptomycin 1g daily im; Tetracycline 2g daily (0.5mg/6 hours), Rifampicin: 600-1200mg daily or 10-20 mg/Kg/day; TMP/SMX between 160/800 mg /8 hours and 80/400 mg /12 hours or trimetroprim 8-10 mg/Kg/day and sulfametoxazole 40-50mg/Kg/day; Ciprofloxacin: 500-1000 mg /12 hours; Ofloxacin: 400mg daily; Ceftriaxone 2-4g/day.
