# Supplementary material for: Systematic Review and Meta-Analysis of Randomized Clinical Trials in the Treatment of Human Brucellosis
Source: PLoS One. 2012 Feb 29;7(2):e32090. doi: 10.1371/journal.pone.0032090 (PMC3290537; doi:10.1371/journal.pone.0032090)
Supplement: Text S1 — Review protocol. (DOC) [file pone.0032090.s007.doc]

**TEXT S1: Review protocol**

**Searh strategy**

1.-Inclusion criteria

All randomized controlled trials comparing two or more antibiotic treatment regimens in human brucellosis, with the following characteristics:

1. Diagnosis criteria were well established
2. The treatment administered is well specified with its duration and dosage
3. The follow-up period was a minimum of six moths
4. Studies with patients with acute non-focal human brucellosis
5. Studies with at least 10 patients in both arms

2.-Exclusion criteria

1. Studies with no well specified treatment
2. Congress abstract
3. Observational or other non randomized studies were not included in meta-analysis
4. Commentaries or reviews
5. Duplicate publications from the same study
6. Randomized controlled trials solely with patiens with osteoarticular brucellosis, neurobrucellosis or brucellar endocarditis.

3.-Databases searched

- PubMed
- The Cochrane Central register of Controlled Trials (CENTRAL)
- Related articles
- Hand searching reference lists of identified studies

4.-PubMed search

- Time limit: articles published from 1985 to present
- Language limits: no language restrictions
- Date of the search: January 2011
- Search query: (“brucella” OR “human brucellosis”) AND (“treatment” OR “therapy”) AND “clinical trial”

**Study selection process**

- Search in PubMed and CENTRAL databases 🡪 Remove duplicate records 🡪 Investigators review titles/abstracts 🡪 selection of full text records for eligibility 🡪 selection of records included 🡪 hand searching reference list of included studies 🡪 extraction of data and resolve discrepancies between investigators.

**Parameters considered in the evaluation of the different treatments**

1. Relapses: reappearance of signs or symptoms of the disease or positive culture results after completion of therapy during follow-up, and after an asymptomatic period.
2. Failure of treatment: persistence of signs or symptoms after the beginning of treatment beyond a period considered appropriate in the various studies.
3. Serious side effects: if withdrawal or change of treatment was necessary.
4. Moderate side effect: if withdrawal or change of treatment was not necessary.
5. Number of patients died of brucellosis.

**Quality assessment:**

1. Assessment of sequence generation and allocation concealment (selection bias)
2. Assessment of blinding of participants and personnel (performance bias)
3. Assessment of incomplete outcome data (attrition bias)

Selection bias and attrition bias assessed using a three grade scale (recommended in the Cochrane handbook for systematic reviews of interventions, version 5.1.0, updated in March 2011):

- Low risk of bias
- Unclear risk of bias
- High risk of bias

**Data extraction**

The data extracted for each study was:

1. Year of publication
2. Study type (randomization and blinding)
3. Number of patients treated
4. Diagnostic criteria and exclusion criteria for patients
5. Number of patients lost to follow-up and the reason for it
6. Antibiotic regimens used, with dosage and treatment duration, as well as route of administration
7. Percentage of patients with focal disease in each study
8. Number of relapses and treatment failures
9. Time lapse between the beginning of treatment and the disappearance of fever or symptoms
10. Follow-up period
11. Side effects of medication, with indication of effects requiring treatment withdrawal
12. Mortality
13. Whether patients were admitted to a hospital at the beginning of or during treatment.
